# RNA‐PROTACs: Degraders of RNA‐Binding Proteins

**DOI:** 10.1002/anie.202012330

**Published:** 2020-12-10

**Authors:** Alice Ghidini, Antoine Cléry, François Halloy, Frédéric H. T. Allain, Jonathan Hall

**Affiliations:** ^1^ Department of Chemistry and Applied Biosciences ETH Zurich Vladimir-Prelog-Weg 4 8093 Zurich Switzerland; ^2^ Department of Biology ETH Zurich Hönggerbergring 64 8093 Zurich Switzerland

**Keywords:** Lin28, oligonucleotides, PROTAC, proteasomal degradation, RNA-binding proteins

## Abstract

Defects in the functions of RNA binding proteins (RBPs) are at the origin of many diseases; however, targeting RBPs with conventional drugs has proven difficult. PROTACs are a new class of drugs that mediate selective degradation of a target protein through a cell's ubiquitination machinery. PROTACs comprise a moiety that binds the selected protein, conjugated to a ligand of an E3 ligase. Herein, we introduce RNA‐PROTACs as a new concept in the targeting of RBPs. These chimeric structures employ small RNA mimics as targeting groups that dock the RNA‐binding site of the RBP, whereupon a conjugated E3‐recruiting peptide derived from the HIF‐1α protein directs the RBP for proteasomal degradation. We performed a proof‐of‐concept demonstration with the degradation of two RBPs—a stem cell factor LIN28 and a splicing factor RBFOX1—and showed their use in cancer cell lines. The RNA‐PROTAC approach opens the way to rapid, selective targeting of RBPs in a rational and general fashion.

## Graphical abstract

RNA‐PROTACs are new chimeric structures that mediate the proteasomal degradation of RNA‐binding proteins (RBP). They comprise an E3‐recruiting peptide conjugated to a short modified RNA, which is iso‐sequential with the native binding element to which the RBP binds in cells. RNA‐PROTACs offer a fast, rational and general approach to target an entire class of protein (RBPs) that has proven difficult until today to target pharmacologically.

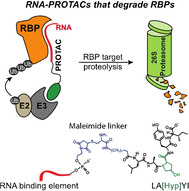

## Introduction

RNA binding proteins (RBPs) constitute a large fraction of a cell's proteome.^[1]^ Over 1500 RBPs are known and their genes are evolutionally conserved and transcribed into splicing variants with unique functions. RBPs bind to RNAs in a dynamic, coordinative and sequence‐selective manner to form ribonucleoprotein (RNP) complexes that play key roles in RNA‐dependent processes.^[2]^ Several diseases are caused by genetic alterations in RBPs, which affect their binding to RNA.^[3]^

Here we introduce a new concept in targeting RBPs using a novel class of chimeric structure, which we have termed RNA‐PROTACs. Conventional PROTACs (PROteolysis‐TArgeting Chimeras) comprise three components: a target‐binding ligand, a ubiquitin E3 ligase‐recruiting ligand and a connecting linker group.^[4]^ The function of the E3 binding moiety is to recruit E3‐dependent factors, whereby formation of a ternary complex between the PROTAC and the protein of interest (POI) via the targeting ligand, activates ubiquitination of the latter, marking it for degradation through the proteasome pathway.^[5]^ This results in lowered levels of the target, and a corresponding loss of its function. Following a rapid phase of development, the first PROTACs have now entered clinical trials.^[6]^

The targeting ligand of a conventional PROTAC is a drug‐like small molecule that binds selectively to the POI. Ligands have been reported for a variety of proteins, including enzymes and receptors.^[4]^ They are typically identified by high‐throughput screening or rational design, based upon the natural ligands of the target proteins.^[7]^

Intuitively, the RNA binding site of an RBP represents a viable target site for drugs, since it is functionally important and structural data for RBPs is often available. However, with the exception of a few natural products inhibiting splicing, (ref. [8] and refs therein) examples of small‐molecule ligands that target RBPs are rare.^[9]^ This may be due to several reasons: RBP binding pockets are intrinsically disordered and change conformation upon RNA binding;^[10]^ compound‐screening assays are challenging to implement;^[11]^ and RBPs share homologous domains that recognize short, degenerate sequence motifs.^[12]^

**Figure 1:**
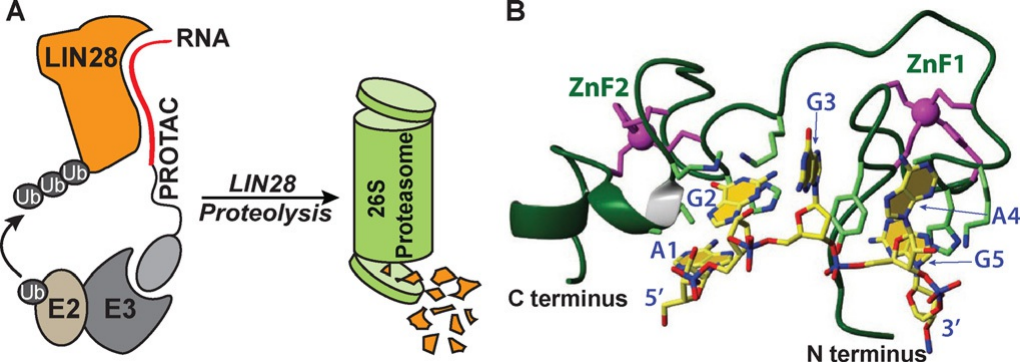
RNA‐PROTACs bind RBPs and direct them for degradation. A) An RNA‐PROTAC comprising a short oligonucleotide binds the RNA‐binding domain of the RBP and mediates its ubiquitination and degradation. B) The Lin28 zinc finger domain binding to its consensus sequence AGGAGAU (adapted from Ref. [13b], PDB: 2LI8).

We employed short oligonucleotides that are iso‐sequential with the RNA consensus binding element (RBE) of an RBP for the first time as the targeting moiety for the PROTAC (Figure 1 A). The oligonucleotide competes with native RNAs for binding to the RBP in cells. We describe here a proof‐of‐concept for this approach with the design, synthesis and characterization of an RNA‐PROTAC targeting LIN28 (Lin28A), a small RBP comprising a C‐terminal CCHC‐type zinc knuckle domain (ZKD).^[13]^

To target Lin28, we used 5′‐AGGAGAU‐3′ (L28_RBE_), a conserved sequence present in microRNAs, to which Lin28 binds (Figure 1 B). We modified L28_RBE_ with 2′‐*O*‐methoxyethyl (MOE) ribonucleotides and conjugated it to a E3‐recruiting peptide derived from the HIF‐1α protein. This RNA‐PROTAC binds selectively to Lin28 in vitro, and suppressed levels of Lin28A in two cancer cell lines in ubiquitin‐dependent fashion. Taken together, these findings pave the way to a new class of rationally designed inhibitors for RBPs, a protein family that until now has been difficult to address with conventional drugs.

## Results and Discussion

A short oligoribonucleotide corresponding to the consensus RBE was a conceptually natural choice for the targeting element of an RNA PROTAC. Short oligonucleotides have been investigated previously as antisense^[14]^ and anti‐miRNA reagents,^[15]^ but have not been pursued into clinical development to our knowledge. Oligoribonucleotides require structural modifications to protect them against ubiquitous nucleases in vivo. Therefore, we adopted a similar strategy employed for recently approved oligonucleotide therapeutics: replacement of the phosphodiester backbone by diastereoisomeric phosphorothioate (PS) linkages^[16]^ and alkylation of the 2′‐hydroxyl group. PS‐linkages endow oligonucleotides with three essential properties for use in vivo: enhanced nuclease resistance, an ability to enter cells and a weak non‐specific binding to proteins that slows renal excretion and allows systemic circulation.^[17]^ Especially, the alkylation of the ribose 2′‐OH with 2′‐*O*‐methoxyethyl (MOE) substituents^[18]^ brings further nuclease stability,^[19]^ and adds a hydration layer to the structure that improves biodistribution and tolerability in vivo.^[20]^

**Figure 2:**
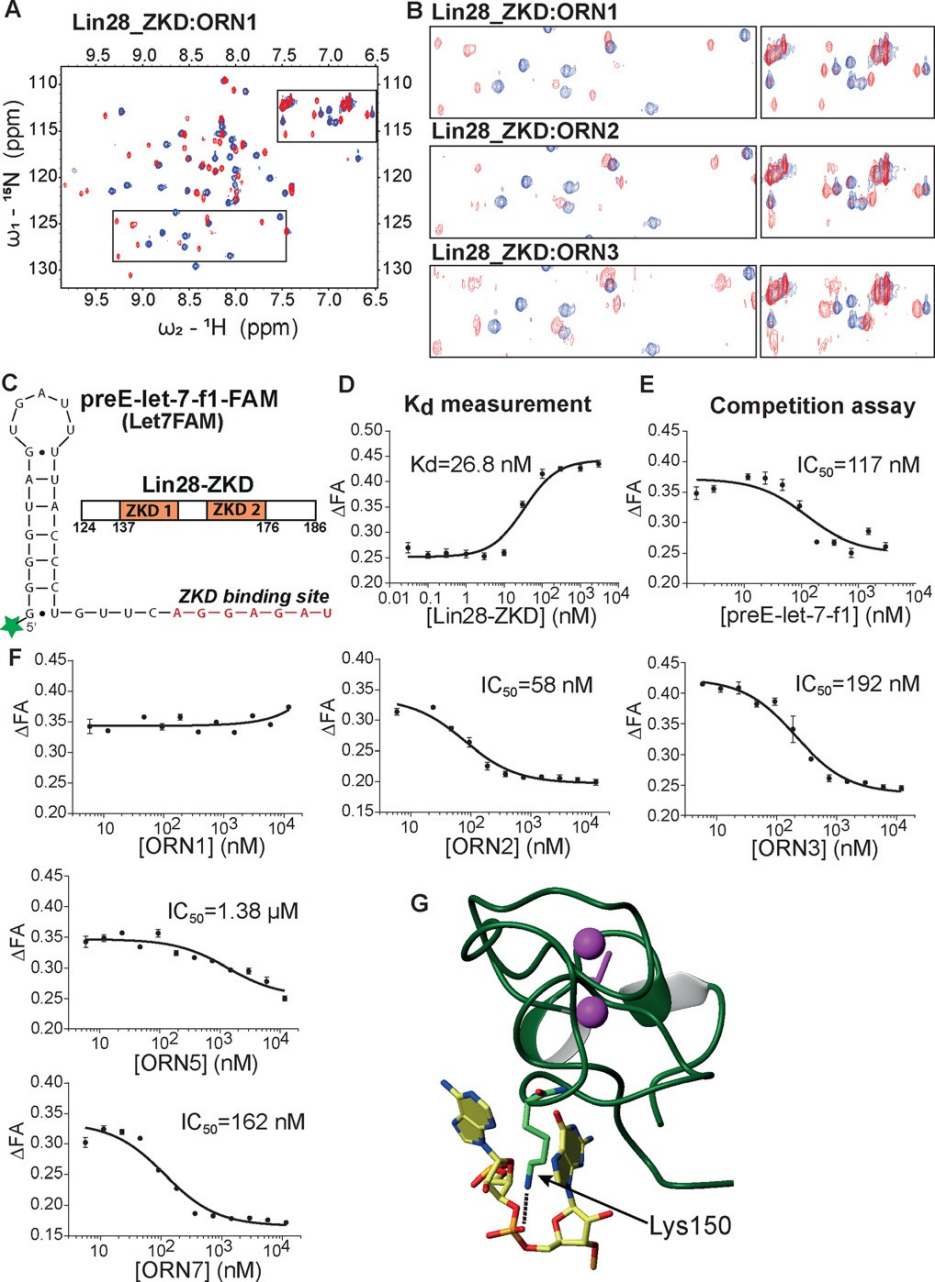
Fluorescence polarization competition assay with Lin28‐binding RNA mimics. A) ^1^H–^15^ N HSQC spectra of Lin28_ZKD, free (blue) and bound (red) to **ORN1** at a 1:1 ratio. B) Overlay of selected ^1^H–^15^ N HSQC spectra of Lin28_ZKD, free (blue) and bound (red) to **ORN1**, **ORN2** and **ORN3** at 1:1 ratios. C) Sequence of FAM labelled preE‐let‐7f‐1 used in this study; domain structures of Lin28 (ZKD: orange; RNA residues that bind the ZKD: red). D) Fluorescence polarization assay of Lin28_ZKD binding to preE‐let‐7f‐1‐FAM. E) Competition assay using unlabeled preE‐let‐7f‐1. F) Competition fluorescence polarization assays with **ORN1**, **‐2**, **‐3**, **‐5** and **‐7**. Wild‐type RNA (**ORN1**) is a weak competitor to preE‐let‐7f‐1. (*n*=3 replicates) G) The solution structure of Lin28_ZKD bound to 5′‐AGGAGAU‐3. Dotted line identifies possible interaction between Lys150 and the phosphorothioate linkage between residues A_4_/G_5_ (adapted from Ref. [13b], PDB: 2LI8).

The oligonucleotide element of an RNA‐PROTAC binds its target RBP via interactions at the riboses and the backbone, as well as the nucleobases. Therefore, it was important to show first that the MOE and PS groups would not disturb these interactions. We showed previously using NMR spectroscopy that Lin28_ZKD recognizes the Watson–Crick faces of G_2_ and G_5_ in 5′‐A_1_G_2_G_3_A_4_G_5_A_6_U_7_‐3′ (Figure 1 B), and that the two zinc finger domains are necessary and sufficient to recognize selectively the GNNG sequence of its partner RNAs.[^13b^, ^21^] Thus, we used the same method to examine how various ribose modifications of L28_RBE_ (Table 1, Figure S1) might affect binding of an RNA‐PROTAC to Lin28_ZKD. Pleasingly, we observed similar chemical shift perturbations for these analogs as for wild‐type RNA **ORN1** (Figure 2 A). This suggested that the modified oligonucleotides adopted a similar mode of binding to Lin28_ZKD. In the case of **ORN2**, **ORN3** and **ORN7** (Figure 2 B, Figure S2) some of the NMR signals broadened or disappeared (precipitation at higher concentrations was also observed). This may have been at least partly caused by the presence of PS diastereoisomers.

**Table 1:** Oligonucleotide sequences used in this study and corresponding *IC*_50_ values determined by fluorescence polarization (**ORN5** is a randomized negative control sequence).

Name	L28_RBE_‐containing sequences (5′ to 3′)	Backbone chemistry	*IC*_50_ [nM]
**Let7_FAM_**	FAM_GGGGUAGUGAUUU UACCCUGUUCAGGAGAU	PO‐RNA	117.4±17.9
**ORN1**	AGGAGAU	PO‐RNA	n.d.
**ORN2**	AGGAGAU	PS‐RNA	58.1±19.8
**ORN3**	AGGAGAU	PS‐MOE	192.2±34.8
**ORN4**	AGGAGAU	PS‐OMe	55.4±13.3
**ORN5**	UUUAUUG	PS‐MOE	1384.5±268.0
**ORN6**	AGGAGAU	PO‐OMe	–
**ORN7**	AGGAGAUAACU	PS‐MOE	162±38
**ORN8**	AGGAG	PS‐MOE	1289±339.4

[a] n.d.=not determined; ORN6 was not measured.

In cells, RNA‐PROTACs must compete with native RNAs for binding the target RBP. In general, RNA‐RBP binding interactions are complex (sometimes multimeric) and often extend outside the RBE.^[12]^ RNA secondary structure may also facilitate the interactions. Therefore, in order to determine whether short, modified derivatives of L28_RBE_ could compete with native RNAs for binding to Lin28 in cells, we adapted a competition binding assay that was originally developed to screen for small‐molecule inhibitors of Lin28 (Figure S3).[^13c^, ^22^]

PreE‐let‐7f‐1 RNA is a 30‐nucleotide (nt) stretch of structured sequence present in let‐7 precursors to which Lin28_ZKD binds with nanomolar affinity.[^13c^, ^22^] Thus, preE‐let‐7f‐1 was synthesized with a 6‐fluoroscein (FAM) label at its 5′ terminus (let7_FAM_; Figure S1, Figure 2 C). It was then incubated with graded concentrations of human recombinant Lin28_ZKD^[13b]^ in order to determine an equilibrium dissociation constant for the Lin28_ZKD/let7_FAM_ interaction. This yielded a *K*_D_ of 27 nM, in agreement with previously recorded values^[22]^ (Figure 2 D). Based on this, 40 nM concentrations of Lin28_ZKD were subsequently used for testing L28_RBE_ analogs.

Incubation of Lin28_ZKD/let7_FAM_ with unlabeled preE‐let‐7f‐1 decreased the fluorescence polarization signal with an *IC*_50_ of 117 nM (Figure 2 E), consistent with displacement of let7_FAM_ from the protein. In contrast, the 7‐mer **ORN1** (unmodified L28_RBE_ RNA) was inactive (Figure 2 F), consistent with observations of Wang et al.^[22]^ The stark difference between the activities of the 7‐mer and the 30‐mer RNAs in this assay may indicate that Lin28_ZKD can make contacts with elements outside of the 7‐nt consensus sequence in native RNAs. Surprisingly, the fully phosphorothioated analog **ORN2** yielded an *IC*_50_ of 58 nM in the assay (Figure 2 F). **ORN4** and **ORN3**, which have PS‐OMe and PS‐MOE modifications, respectively, yielded *IC*_50_ values of 56 and 192 nM, respectively. A randomized sequence (negative control) of L28_RBE_ (**ORN5**) and a 5‐nt analog of **ORN3** (**ORN8**) showed about 20‐fold and 7‐fold weaker activities than **ORN4** and **ORN3**, respectively.

To provide insight on why the short PS‐oligonucleotides displaced let7_FAM_ so readily from Lin28_ZKD, we measured binding affinities of selected PO‐ and PS‐ L28_RBE_ analogs to Lin28_ZKD by surface plasmon resonance spectroscopy (SPR). Lin28_ZKD was immobilized to a chip and incubated with graded concentrations of selected ORNs, as well as positive and negative controls.

A let‐7 precursor, pre‐let‐7 g bound Lin28_ZKD with the highest affinity (*K*_d_ approx. 10 nM; Figure S4). **ORN1**, **ORN2** and **ORN3** bound Lin28_ZKD with *K*_d_s of 25.8 μM, 550 nM and 580 nM, respectively. We re‐examined the NMR‐structure of Lin28_ZKD/L28RBE in an effort to explain the enhanced binding/activity of the PS‐oligonucleotides. Interestingly, the phosphodiester group between A_4_/G_5_ of **ORN1** contacts Lys_150_ of Lin28_ZKD (Figure 2 G). Thus, a phosphorothioate group at this position might positively enhance this interaction and partly explain the superior activity of the PS‐analogs. Based on the results from these independent assays, three L28_RBE_ analogs (**ORN3**, **ORN4** and **ORN7**), together with the sequence‐randomized control (**ORN5**) were selected for further study.

Various E3 systems have been employed as PROTACs to mediate degradation of POIs.[^5c^, ^23^] Empirical testing is generally used to identify that combination of linker and E3 system that yields the most potent PROTAC reagent.^[24]^ One of the earliest PROTAC systems was an ALAPYIP‐containing peptide derived from the HIF‐1α (hypoxia‐inducible factor 1α) transcription factor. When its central proline is hydroxylated,^[25]^ this peptide recruits VHL (von Hippel‐Lindau) into the VBC‐Cul2 E3 ubiquitin ligase complex.^[26]^ A shortened segment of the peptide (comprising the hydroxylated proline; LA[Hyp]YI) still retains ubiquitinating activity and was selected for this PoC study, although recent work has uncovered non peptide‐based E3‐ligands that might also be used.^[27]^ LA[Hyp]YI is cell‐permeable,^[28]^ although it seemed likely that the uptake of RNA‐PROTACs into cells would be dominated by the properties of the oligonucleotide.

Single‐stranded PS oligonucleotides are internalized after prolonged incubation into many types of cultured cells via gymnosis. Uptake leads to endosomal‐lysosomal accumulation, with a slow leakage into the cytosol, from where the oligonucleotides distribute to other parts of the cell, including the nucleus.^[29]^ The PS‐groups are crucial for the uptake, and the most efficient uptake is typically seen with longer oligonucleotides.^[30]^ We examined the uptake of FAM‐conjugated oligonucleotides **ORN3** (7‐nt) and **ORN7** (11‐nt) into human immortalized myelogenous leukemia line K562 cells using fluorescence microscopy. Both **ORN3** and **ORN7** were visible in the cytosol and the nuclei of cells, with the uptake of the longer **ORN7** being higher than that of **ORN3** (Figure S5). Taken together, the data suggested that an RNA‐PROTAC comprising a 7‐mer PS‐MOE sequence would reach the cell cytoplasm and therefore be able to elicit its effect.

**Figure 3:**
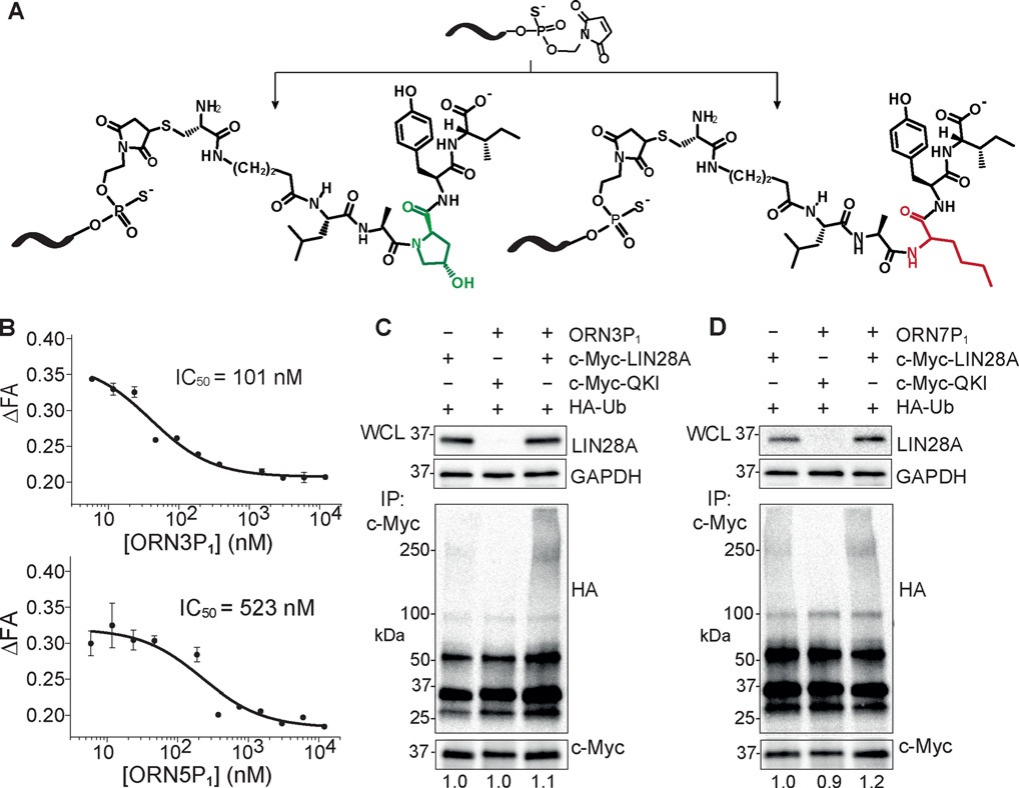
Synthesis of RNA‐PROTACs and their mechanism of action. A) Synthetic route to RNA‐PROTACs and controls. B) Fluorescence polarization assay for **ORN3P_1_** and negative seq‐control **ORN5P_1_**. C,D) RNA‐PROTAC‐mediated ubiquitination of myc‐Lin28A in 22Rv1 cells co‐transfected with HA‐Ubiquitin (HA‐Ub) and pCMV‐myc‐Lin28A, or pDEST‐Myc‐QKI with either vehicle (OptiMEM) or 500 nM **ORN3P_1_** (C) or **ORN7P_1_** (D). cMyc‐immunoprecipitated lysates were separated by SDS‐PAGE followed by western blots detecting HA (Ub). Slow‐migrating smears in right‐hand lanes represent ubiquitin‐conjugated cMyc‐Lin28A. WCL=whole‐cell lysate input.

L28_RBE_ analogs (**ORN3**, **ORN4**, **ORN5**, **ORN7**) were conjugated in solution to LA[Hyp]YI via the peptide N‐terminus (Figure 3 A, Figures S1, S6). The PS‐MOE derivative **ORN3** was prioritized for study since PS‐MOE oligonucleotide drugs distribute widely after administration in vivo,^[17b]^ are metabolically stable and are safe for patients.^[31]^ Masked maleimide‐oligonucleotides were prepared on solid support. They were activated prior to coupling with peptide P_1_ (LA[Hyp]YI) or a control sequence P_con_, in which norleucine was exchanged for the hydroxyproline (Figure 3 A). In all, ten oligonucleotide‐peptide conjugates were synthesized and purified (Table 2, Figure S1). Several of the conjugates were tested in the Lin28_ZKD competition binding assays to ensure that the addition of the linker/peptide did not adversely affect binding of the RNA‐PROTAC to Lin28 (Figure 3 B, Figure S7). **ORN3P1** and **ORN7P1** competed slightly more strongly for binding to Lin28_ZKD than their parent unconjugated oligonucleotides, presumably due to contacts between LA[Hyp]YI and the protein (Figure 3 B, Figure S7). Consistent with this, the sequence control ORN5P1 showed a weak effect on Lin28_ZKD (Figure S7).

**Table 2:** RNA‐PROTACs synthesized and used in this study.

Name	Sequence	Peptide^[a]^	Mass obs. [g mol^−1^]	Mass calc. [g mol^−1^]
**ORN3P_1_**	AGGAGAU	C[Ahx]LA[Hyp]YI	3810.8	3811.5
**ORN5P_1_**	UUUAUUG	C[Ahx]LA[Hyp]YI	3742.8	3743.5
**ORN3P_con_**	AGGAGAU	C[Ahx]LA[Nle]YI	3810.8	3811.6
**ORN4P_1_**	AGGAGAU	C[Ahx]LA[Hyp]YI	3488.5	3489.2
**ORN4P_con_**	AGGAGAU	C[Ahx]LA[Nle]YI	3488.5	3489.2
**ORN7P_1_**	AGGAGAUAACU	C[Ahx]LA[Hyp]YI	5404.9	5404.2
**ORN7P_con_**	AGGAGAUAACU	C[Ahx]LA[Nle]YI	5404.9	5404.3
**ORN9P_1_**	UGCAUGU	C[Ahx]LA[Hyp]YI	3766.7	3767.5
**ORN10P_1_**	GCCAUCU	C[Ahx]LA[Hyp]YI	3739.8	3740.5
**ORN9P_con_**	UGCAUGU	C[Ahx]LA[Nle]YI	3766.8	3767.6

[a] Ahx=6‐(Fmoc‐amino)hexanoic acid, Nle=Norleucine, Hyp=l‐hydroxyproline (mutated negative control peptide).

Next, we investigated the ability of **ORN3P_1_** and **ORN7P_1_** to mediate ubiquitination of Lin28 in cells, using a previously described protocol.^[32]^ Cells from the prostate cancer cell line 22RV1, which express very low levels of Lin28, were co‐transfected with two plasmids expressing pCMV‐myc‐Lin28A and HA‐tagged polyubiquitin (HA‐Ub).

After 48 h of incubation with **ORN3P_1_** and **ORN7P_1_**, the tagged Lin28 (and the control QKI) was immunoprecipitated using an anti‐Myc antibody. The isolated protein was then analyzed by western blotting using the anti‐HA and anti‐c‐Myc antibodies. Consistent with other previously described PROTAC reagents,[^32^, ^33^] **ORN3P_1_**‐ and **ORN7P_1_**‐treated cells showed a smear on the gel towards higher molecular weight (Figure 3 C,D, Figure S8), consistent with conjugation of poly‐Ub to the myc‐Lin28A fusion protein. Vehicle‐treated cells, or cells transfected with a plasmid that expresses an alternative RBP as negative control (Quaking homolog, QKI; pDEST‐Myc‐QKI) instead of pCMV‐myc‐Lin28A, showed no such effects.

**Figure 4:**
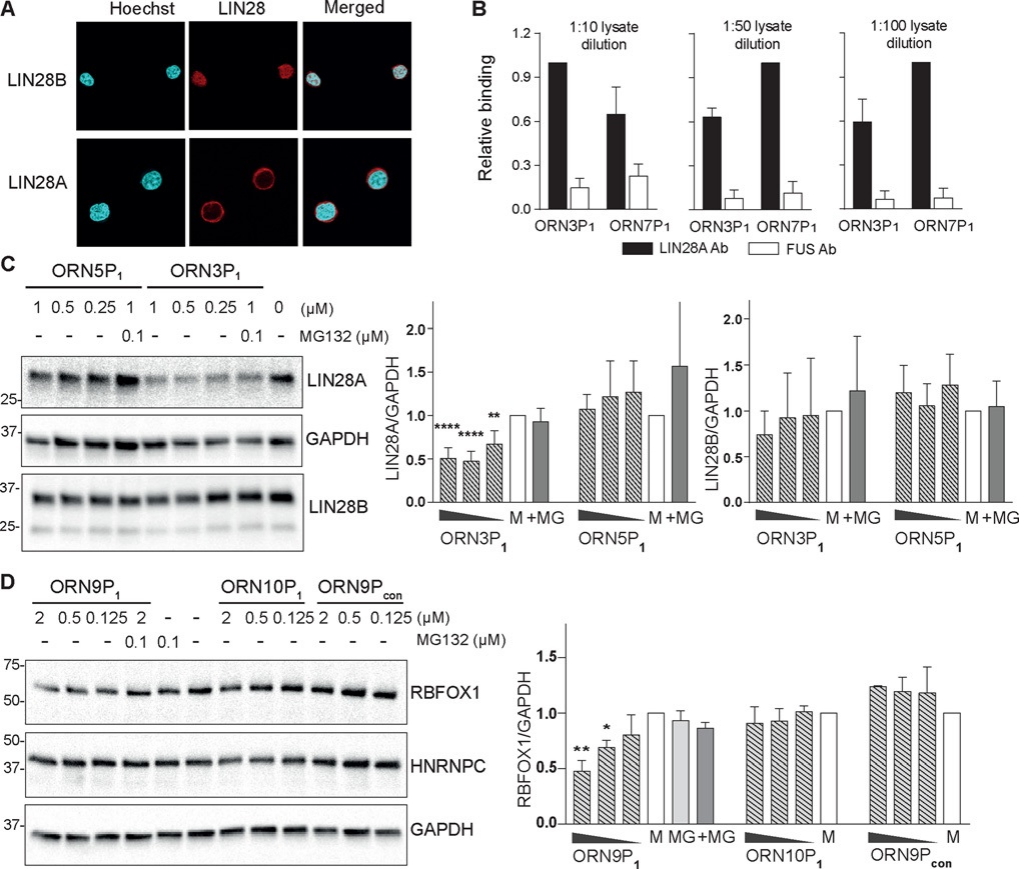
RNA‐PROTACs mediate Lin28A and RBFOX1 degradation in cells. A) Lin28A and Lin28B localization in K562 cells; Hoechst staining indicates location of nuclei. B) Pull‐down of Lin28A onto streptavidin‐coated microtiter plates from the lysates of K562 cells treated with biotin‐labeled **ORN3P1** and **ORN7P1**. Relative binding is normalized to mock‐treated cells. C) Western blot and quantification of Lin28A from K562 cells incubated with **ORN3P_1_** and negative seq‐control **ORN5P_1_** for 48 h. D) RNA‐PROTACs mediate degradation of RBFOX1 in HEK293 cells. HEK293 cells were incubated with **ORN9P_1_**, **ORN10P_1_** and negative peptide‐control **ORN9P_con_** for 48 h. Data are shown as mean ±SD of three independent experiments for (C) and two for (D). M=cells treated with mock solutions, MG=cells treated with 100 nM MG132, +MG=cells co‐treated with RNA‐PROTACs and 100 nM MG132. Error bars indicate standard deviations. Asterisks denote statistical significance compared to 0 nM dose assessed by 1‐way ANOVA test whereas: ns *P*>0.05, * *P*≤0.05, ** *P*≤0.01, *** *P*≤0.001, **** *P*≤0.0001. Uncropped western blots are shown in Figure S14,15.

Humans express two paralogs of the protein, LIN28 (Lin28A) and LIN28B (Lin28B), that play key roles in development, metabolism, and pluripotency.^[34]^ Lin28 proteins are abundantly expressed during embryonic development, as well as in several tumors and tumor‐derived cell lines.^[35]^ They bind with nanomolar affinity to let‐7 precursors and the 3′ untranslated regions (UTRs) of mRNAs involved in cell proliferation. In order to confirm that RNA‐PROTACs bind to endogenous Lin28 in cells, we performed a pull‐down RNA ELISA assay that we have previously described.^[36]^ Thus, we conjugated a biotin group to the 3′ ends of **ORN3P_1_** and **ORN7P_1_** and added these reagents separately on to myelogenous leukemia K562 cells. We incubated lysates from treated cells on streptavidin‐coated plates so as to capture the RNA‐PROTACs present in the cell lysates. We then assayed for the presence of Lin28A in the plates using an anti‐Lin28A antibody and a negative control anti‐FUS antibody. Lysates from both **ORN3P_1_**‐ and **ORN7P_1_**‐treatments yielded a strong signal for Lin28A protein, in comparison to lysates from mock‐treated cells, or compared to measurement using the FUS antibody (Figure 4 B, Figure S9). The data confirmed that RNA‐PROTACs entered cells and engaged their intended target.

Finally, we tested RNA‐PROTACs for their ability to degrade their target in two cell lines: the embryonic NT2/D1 cell line, which expresses high levels of Lin28A,^[13a]^ and K562 cells which express Lin28A and Lin28B. NT2/D1 cells treated with 2 μM concentrations of **ORN3P_1_** or **ORN7P_1_** showed an approximate 50 % reduction of Lin28A, whereas the negative control peptide **ORN3P_con_** was inactive (Figure S10). Suppression of the target was not quantitative, as is often observed from testing PROTAC reagents without an optimization of the targeting ligand, the linker and the E3 system. Furthermore, there was no statistical difference in the activity of the 7‐mer and 11‐mer reagents. In K562 cells, **ORN3P_1_** suppressed 50 % of Lin28A at the highest concentration, whereas the negative control **ORN5P_1_** was inactive, confirming a sequence‐selective action of the RNA‐PROTAC (Figure 4 C). The reagents had no discernible effect on cell toxicity (Figure S11). Lin28A and Lin28B have identical zinc finger domains,[^13c^, ^21^] however **ORN3P_1_** had little effect on Lin28B (Figure 4 C), despite its presence in the nucleus. This may have been in part, because Lin28B is mainly localized in the nucleus where proteasomal degradation might be less effective or require additional steps or factors (Figure 4 A, Figure S12).[^35b^, ^37^] Finally, we co‐treated K562 cells with **ORN3P_1_** and MG132, a reversible inhibitor of the 26S proteasome. This largely attenuated Lin28A suppression by the RNA‐PROTAC, strongly suggesting that **ORN3P_1_** elicited its effects through the proteasome, as intended (Figure 4 C). In order to explore the versatility of RNA‐PROTACs with non peptide‐based VHL ligands, we conjugated small‐molecule VH032^[38]^ to **ORN3** via an amino linker to yield **ORN3VH_032_** (Figure S1). In K562 cells, **ORN3VH_032_** also reduced levels of Lin28A after 24 h in a way that could be rescued by addition of MG132 (Figure S13).

In order to demonstrate that RNA‐PROTACs can be generated against other RBPs using their respective consensus RNA binding elements as a targeting ligand, we turned to RBFOX1. RBFOX1 (A2BP1)^[39]^ is an alternative splicing factor expressed in neuronal tissues,^[40]^ muscle and heart.^[41]^ It binds with nanomolar affinity to 5′‐UGCAUGU‐3′ through its RNA recognition motif (RRM). We synthesized a PS‐MOE‐modified variant of the RBFOX1 RBE (FOX_RBE_) and conjugated it to C[Ahx]LA[Hyp]YI (**ORN9P_1_**) using the same protocol as for the Lin28 RNA‐PROTACs. In parallel, we prepared two negative control reagents: **ORN10P_1_** comprises a random oligonucleotide sequence, whereas **ORN9P_con_** has the FOX_RBE_ conjugated to the mutated peptide (Table 2).

Human embryonic kidney cells (HEK293T) cells were treated with the three reagents at increasing concentrations, under similar conditions for the Lin28 RNA‐PROTACs. Western blotting of protein isolated from treated cells showed an approximate 50 % reduction of RBFOX1 protein at 2 μM (Figure 4 D). **ORN9P_con_** and **ORN10P_1_** did not affect levels of RBFOX1 protein, suggesting that **ORN9P_1_** is selective for its target. In order to demonstrate further the selectivity of **ORN9P_1_** for RBFOX1, the blot was probed with an antibody against heterogeneous nuclear ribonucleoproteins HNRNPC. HNRNPC contains an amino‐terminal sequence that is known to bind strongly uridine (U)‐rich sequences, and therefore provided a strict test of selectivity for the FOX_RBE_ analogs.^[42]^ Happily, none of the treatments with **ORN9P_1_**, **ORN10P_1_** and **ORN9P_con_** affected significantly the level of endogenous HNRNPC protein (Figure 4 D).

## Conclusion

Conventional drugs can be classified into distinct structural types, such as small‐molecules, therapeutic proteins and oligonucleotides. Single‐stranded oligonucleotide drugs have cellular RNA as their common target, with which they hybridize according to Watson–Crick rules. Amongst them, the most prevalent groups are the antisense‐ and splice‐switching‐oligonucleotides that target mRNAs. However, new targeting mechanisms mediated by anti‐miRNA and decoy oligonucleotides are also under investigation (see refs. [43] and [44] for examples).

Here we describe RNA‐PROTACs, a new class of chimeric oligonucleotides that are rationally designed to target RBPs, that is, proteins. Many groups have conjugated oligonucleotides to peptides, usually however, for the peptide to transport the oligonucleotide to its target RNA in vitro/vivo.^[45]^ Here, the roles of the peptide and the oligonucleotide are reversed; the oligonucleotide delivers the peptide to its target site. RNA‐PROTACs dock into the RNA binding site of an RBP via the structurally modified oligoribonucleotide that is sequence‐identical with the native RNA‐binding element of the RBP. The first RNA‐PROTAC targets the Lin28 protein, a stem cell factor and oncoprotein of high interest as a potential drug target for several diseases. Using a structure‐based approach, we designed a 7‐nt PS‐MOE oligonucleotide analogue that binds tightly to the zinc finger domain of Lin28A. A VHL‐recruiting peptide that is conjugated to the 5′‐end of the oligonucleotide then mediates degradation of the target in cells via the ubiquitination pathway. This proof‐of‐concept represents a new means of degrading and thereby inhibiting RNA‐binding proteins, a target class that until now has proven difficult to address pharmacologically.

## Conflict of interest

The authors declare no conflict of interest.

## Supporting information

As a service to our authors and readers, this journal provides supporting information supplied by the authors. Such materials are peer reviewed and may be re‐organized for online delivery, but are not copy‐edited or typeset. Technical support issues arising from supporting information (other than missing files) should be addressed to the authors.

Supplementary
