# Supplementary material for: RNA‐PROTACs: Degraders of RNA‐Binding Proteins
Source: Angew Chem Int Ed Engl. 2020 Dec 10;60(6):3163–9. doi: 10.1002/anie.202012330 (PMC7898822; doi:10.1002/anie.202012330)
Supplement: Supplementary file 1 — Supplementary [file ANIE-60-3163-s001.pdf]

Angewandte Chemie
Eine Zeitschrift der Gesellschaft Deutscher Chemiker

Supporting Information

RNA-PROTACs: Degraders of RNA-Binding Proteins

*Alice Ghidini, Antoine Cléry, François Halloy, Frédéric H. T. Allain, and Jonathan Hall**

anie_202012330_sm_miscellaneous_information.pdf

Author Contributions

J.H. Conceptualization: Supporting; Funding acquisition: Lead; Project administration: Lead; Supervision: Lead; Writing—original draft: Equal; Writing—review & editing: Equal
A.G. Conceptualization: Lead; Data curation: Lead; Formal analysis: Lead; Methodology: Lead; Supervision: Equal; Validation: Lead; Visualization: Lead; Writing—original draft: Lead; Writing—review & editing: Lead
A.C. Formal analysis: Supporting; Investigation: Supporting; Visualization: Supporting
F.H. Formal analysis: Supporting; Validation: Supporting; Visualization: Supporting; Writing—original draft: Supporting; Writing—review & editing: Supporting
F.A. Data curation: Supporting; Supervision: Supporting; Writing—original draft: Supporting.

Table of contents

Material and methods 2
Fluorescence-activated flow cytometry (FACS). 4
Figure S1. LCMS chromatograms of oligonucleotides and RNA-PROTACS. 14
Figure S2. ^{1}H-^{15}N HSQC spectra of Lin28_ZKD and oligonucleotides. 15
Figure S3. Lin28 fluorescence competition assay. 16
Figure S4. Surface Plasma Resonance of Lin28_ZKD and oligonucleotides. 17
Figure S5. Intracellular uptake of FAM-labelled oligonucleotides and RNA-PROTAC ORN3P_1 18
Figure S6. Solid-phase synthesis of RNA-PROTACs and their controls. 18
Figure S7. Fluorescence polarization assay for RNA-PROTACs ORN3P_1, ORN7P_1 and ORN4P_1. 19
Figure S8. RNA-PROTAC-mediated ubiquitination of myc-Lin28A in 22Rv1 cells (replicate). 19
Figure S9. Pull-down of Lin28A by RNA-PROTACs from K562 cells. 20
Figure S10. Western blot membranes for Lin28A and GAPDH detection in NT2.D1 cells 21
Figure S11. Cytotoxicity of RNA-PROTACs in K562 cells by fluorescence-activated flow cytometry (FACS). 22
Figure S12. Confocal microscopy of K562 for cellular localization of LIN28 and the action of ORN3P_1. 23
Figure S13. ORN3VH_{032} treatment of K562 cells: western blot membranes for LIN28A and GAPDH detection 24
Figure S14. Uncropped western blot membranes for Lin28A, Lin28B, GAPDH detection in K562 cells. 25
Figure S15. Uncropped western blot membranes for RBFOX1, HNRNPC, GAPDH detection in HEK293 cells 26

Material and methods

Synthesis of oligonucleotide sequences. Chemicals for oligonucleotide synthesis were purchased from Aldrich and TCI (Sigma-Aldrich Chemie GmbH, D-89555 Steinheim) and phosphoramidites were obtained from Thermo Fisher Scientific (Waltham, MA), Glen Research (Sterling, VA). The activator 5-benzylthiotetrazole (BTT) was purchased from Biosolve (5555 CE Valkenswaard, the Netherlands). All oligonucleotides used in this work were synthesized with a MM12 synthesizer from Bio Automation Inc. (Plano, TX) on 500Å or 2000 Å UnyLinker CPG from ChemGenes (Wilmington, MA). The coupling time for phosphoramidites was 2x90 s. The RNA phosphoramidites were prepared as 0.08M solutions in dry acetonitrile (ACN), the activator BTT (Biosolve BV, 5555 CE Valkenswaard, the Netherlands) was prepared as a 0.24 M solution dry ACN, 5-(Benzylthio)-1H-tetrazole (Carbosynth) was used at 0.24 M concentration in dry acetonitrile to activate the phosphoramidites for coupling. Oxidizer was prepared as a 0.02 M I_2 solution in THF/Pyridine/H2O (70:20:10, w/v/v/v); the sulfurizing reagent was prepared as a 0.05 M solution of 3-((N,N-dimethylaminomethylidene)amino)-3H-1,2,4-dithiazole-5-thione (DDTT; Sulfurizing Reagent II; Glen Research, Virginia) in dry pyridine/ACN (60:40). Capping reagent A was: THF/lutidine/acetic anhydride (8:1:1) and capping reagent B was: 16% N-methylimidazole/THF. Deblock solution was prepared as a 3% dichloro acetic acid in dichloromethane. The cleavage, the bases and phosphordiester backbone deprotection was done by incubation of the CPG-support for 45 min, at 65 °C in AMA solution (conc. ammonia:methylamine=1:1). Deprotection of 2'-O-TBDMS (tert-butyldimethylsilyl-) was performed by incubation of the oligonucleotides for 1.5 h, at 70 °C in a mixture of dry 1-N-methyl-2-pyrrolidone/triethylamine/triethylamine trihydrofluoride (6/3/4). The oligonucleotides were purified DMT-on and DMT-off by RP-HPLC on an Agilent 1200 series preparative HPLC fitted with a Waters XBridge OST C-18 column, 10x50 mm, 2.5 µm at 60 °C. Running buffer for HPLC purification of single-stranded ORNs (up to 23 nt): buffer A (0.1 M triethylammonium acetate), buffer B (acetonitrile); gradient for the DMT-on purification: 20–50% buffer B over 5min; gradient for the DMT-off purification: 5 –35% buffer B over 5min. Fractions containing the product were collected and dried in a miVac duo SpeedVac from Genevac, diluted with water to 20 µM concentration. Mass and purity (>95%) was confirmed by LCMS (Agilent 1200/6130 system) on a Waters Acquity OST C-18 column, 2.1x50 mm, 1.7 mM, 65 °C. Buffer A: 0.4M HFIP, 15mM triethylamine; buffer B: MeOH. Gradient: 7–35% B in 12 min; flow rate: 0.3 ml/min.

Synthesis of RNA-PROTAC constructs (Peptide ligands). Synthesis of the oligonucleotide sequences of the RNA-PROTAC molecules was carried out as described before. A 5'-maleimide-modifier phosphoramidite (10-1938, GlenResearch) was appended on 5' to ORN3, ORN4, ORN5, ORN7, ORN9 and ORN10 sequences. To avoid loss of the maleimide 2,5-dimethylfuran protecting group, the cleavage from support and bases deprotection was done by incubation of the CPG-support at 30°C in conc. aq. ammonia solution overnight. The oligonucleotides were then purified by RP-HPLC on an Agilent 1200 series preparative HPLC fitted with a Waters XBridge OST C-18 column, 10x50 mm, 2.5 µm at 60 °C. Running buffer for HPLC purification of single-stranded oligos: buffer A (0.1 M triethylammonium acetate), buffer B (acetonitrile); gradient for the purification: 10–35% buffer B over 8min. Fractions containing the product were collected and dried in a miVac duo SpeedVac from Genevac, diluted with water to 20 µM concentration. Mass and purity (>95%) was confirmed by LCMS (Agilent 1200/6130 system) on a Waters Acquity OST C-18 column, 2.1x50 mm, 1.7 mM, 65 °C. Buffer A: 0.4M HFIP, 15mM triethylamine; buffer B: MeOH. Gradient: 5–50% B in 12 min; flow rate: 0.3 ml/min. Maleimide deprotection was performed by microwave irradiation in water for 90 min at 90 °C. Successively, 5eq of peptide (P_1 or P_2) were added to 300 µM solution of deprotected oligonucleotide. Reaction was vortexed for 1h and then purified by RP-HPLC as previously described. Running buffer for HPLC purification of RNA-PROTAC sequences: buffer A (0.1 M triethylammonium acetate), buffer B (acetonitrile); gradient for the purification: 20–50% buffer B over 12 min.

Synthesis of RNA-PROTAC constructs (VH032 ligand). A 5'-amino-modifier phosphoramidite (10-1905-90, GlenResearch) was appended on 5' to ORN3 sequence. The terminal MMt protection was removed by incubation of the support in deblock solution (3% dichloro acetic acid in dichloromethane) 2x3min, followed by 3 washes with ACN. VH032 amide-PEG3-acid (Tocris 6679, 37.5eq.) and HATU (35.6eq.) were dissolved in 100µL of DMF and, after addition of 3µL of DIEA, agitated for 5 min to preactivate the mixture, then added to the solid support and shaken for 30 min. The support was filtered and washed with DMF and ACN. The cleavage from support and bases deprotection was done by incubation of the CPG-support at at 65 °C in AMA solution (conc. ammonia:methylamine=1:1) for 45min. The oligonucleotides were then purified by RP-HPLC on an Agilent 1200 series preparative HPLC fitted with a Waters XBridge OST C-18 column, 10x50 mm, 2.5 µm at 60 °C. Running buffer for HPLC purification of single-stranded oligos: buffer A (0.1 M triethylammonium acetate), buffer B (acetonitrile); gradient for the purification: 10–35% buffer B over 8min. Fractions containing the product were collected and dried in a miVac duo SpeedVac from Genevac, diluted with water to 200 µM concentration. Mass and purity (>95%) was confirmed by LCMS (Agilent 1200/6130 system) on a Waters Acquity OST C-18 column, 2.1x50 mm, 1.7mM, 65 °C. Buffer A: 0.4 M HFIP, 15 mM triethylamine; buffer B: MeOH. Gradient: 5–50% B in 12 min; flow rate: 0.3 ml/min.

Protein expression and purification. Human Lin28_ZKD construct encoding amino acids 124–186 of human Lin28 referred to as Lin28 ZnF12 (NM_024674; Q9H9Z2) was expressed and purified as previously described[20]. For NMR spectra, His6-tagged Lin28_ZKD was expressed in *Escherichia coli* BL21 (DE3) Codon+ (RIL) cells in minimal M9 medium containing 1 g per liter $^{15}NH_4Cl$ at 25 °C overnight following induction with 0.5 mM IPTG and addition of 0.1 mM ZnCl2. Cells were lysed by sonication in 50 mM Tris-Cl pH 8, 1M NaCl, 2 mM β-mercaptoethanol, 0.1 mM ZnCl2 and Lin28_ZKD was purified by Ni-NTA affinity columns in the same buffer and dialyzed in 50 mM HEPES pH 7.2, 150 mM NaCl, 2 mM β-mercaptoethanol, 0.2 mM $ZnCl_2$.

NMR spectroscopy. NMR spectra were acquired on Bruker 600 MHz spectrometer equipped with a cryoprobe. Data were processed using Topspin 2.1 (Bruker) and analyzed with Sparky (http://cgl.ucsf.edu/home/sparky/). Samples contained ^{15}N labelled Lin28_ZnFs in complex with unlabelled oligonucleotides. NMR titrations were conducted by titration of short

oligonucleotides (0.2 nM) into ^{15}N-labeled Lin28-ZnF12 in 10 mM HEPES pH 7.0, 1.5 mM β-mercaptoethanol, 0.15 mM ZnCl2 and monitored by ^{1}H-^{15}N-HSQC at 303K.

Fluorescence Polarization Assay. Fluorescence polarization titration assay was carried out by titrating Lin28_ZKD protein into FAM-labeled PreE-let-7 at a 10 nM final concentration and buffer (10 mM HEPES, 150 mM NaCl, 10 µM $ZiCl_2$, pH 7,4). The assay plate was briefly vortexed, and air bubbles were removed by spinning at 1000 rpm. The fluorescence anisotropy was measured on a Spectra Max Paradigm multimode plate reader (Molecular Devices). The fluorescence polarization competition assay was carried out in a similar fashion, except compounds were titrated into buffer containing Lin28_ZKD and FAM-labeled preE-let-7. The concentration of Lin28_ ZKD is kept at 40 nM, a value in the range of the dissociation constant of Lin28_ZKD and preE-let-7 determined through fluorescence polarization titration assay, whereas preE-let-7 concentration is kept constant at 10 nM. FP competitive binding assays were performed on 384-well plates (#3575, Corning) with a total well volume of 10 µL. Fluorescence polarization readings were transferred into Prism 7.0 (GraphPad). All fluorescence polarization assays were repeated at least three times and the anisotropy values fitted using GraphPad Prism software using 1:1 binding/inhibition model.

Analysis of RNA-Protein Interactions with SPR Technology (immobilized Lin28_ZKD). Realtime affinity determination of Lin28_ZKD with different oligonucleotides was achieved using a MASS-1 device (Sierra Sensors, Hamburg, Germany). All experiments were performed at 25 °C on nitrilotriacetic acid sensor chip (His-tag series chips from Sierra Sensors, Hamburg, Germany). The chip contains eight flow cells. The signals from a flow cell without protein was used as a reference (the protein immobilization step was skipped). All cells were treated and detected simultaneously. The cells were loaded with 30 µl of loading buffer (200 µM $NiCl_2$ in running buffer: 10 mM HEPES, pH 7.4, 150 mM NaCl, 0.005% Tween 20) at 10 µl/min. After washing the flow cells for 300 s at 10 µl/min with running buffer, Lin28_ZKD protein was injected into flow cells from 50 µg/mL solutions, with 10 µl/min. Protein attachment to the chip surface was manually restricted to yield a mean difference of 1200–1500 RU. Afterward, 100 µl of oligo was injected into all flow cells at 25 µl/min (reference cells as well). The oligonucleotides were injected in at least five different concentrations with a replicate on the same immobilized protein. The eighth channel represent a reference at concentration zero of the oligos. To eliminate the possibility that any RNA potentially still bound to the fixed protein would interfere with subsequent analysis, the chip surface was regenerated after each measurement by applying 20 µl of regeneration buffer (10 mM HEPES, pH 7.4, 150 mM NaCl, 500 mM EDTA, 0.005% Tween 20) at 10 µl/min. Signals from a flow cell without protein were used as reference. Data were evaluated with the Sierra Analyzer software 3.1.14 (Sierra Sensors, Hamburg, Germany), and the obtained sets of sensorgrams were fitted into Langmuir's model ratio of 1:1 binding.

Cell culture and gymnosis of RNA PROTAC. K562, 22Rv1 and NT2/D1 cell lines were purchased from American Type Culture Collection (ATCC) and grown in a humidified incubator with 5% CO_2 at 37°C. Cells were routinely tested for mycoplasma contamination using MycoAlert™ PLUS Mycoplasma Detection Kit (Lonza, Cat. # LT07). K562 cells were cultured in Iscove's Modified Dulbecco's Medium (31966-021, ThermoFisher Scientific) supplemented with 10 % of FBS (fetal bovine serum, 10270106, ThermoFisher Scientific) and 4 mM L-Glutamine. For gymnosis experiments, K562 cells were plated in 24 well/plates (480 000 cells/well), 300 µl volume. Oligonucleotide gymnosis was performed adding to the cells 25 µl of Oligo PROTAC in Opti-MEM (31985-047, Gibco) at different concentrations. After 48 h, the supernatants were removed and protein extracts isolated from the cells.

22Rv1 (ATCC, CRL-2505) cell lines were cultured in RPMI 1640 medium (Corning, Cat. # 10-040-CV) supplemented with 10% fetal bovine serum (Corning, Cat. # 35010CV), as recommended by the supplier. For gymnosis experiments, 22Rv1 cells were plated in 12-well plates (480 000 cells/well), 500 µl volume. Oligonucleotide gymnosis was performed adding to the cells 25 µl of RNA-PROTAC in Opti-MEM (31985-047, Gibco) at different concentrations. After 48 h, the supernatants were removed and protein extracts isolated from the cells.

NT2/D1 human embryonal carcinoma (ATCC, CRL-1973) cells were grown in DMEM (ThermoFisher Scientific) supplemented with 10% FBS as previously described. For gymnosis experiments, NT2/D1 cells were plated in 24-well plates (480 000 cells/well), 300 µl volume. Oligonucleotide gymnosis was performed as described for the previous cells.

HEK293 human embryonic kidney cells (ATCC, CRL-1573) were cultured in DMEM (ThermoFisher Scientific) supplemented with 10% FBS. For gymnosis experiments, HEK293 cells were plated in 24-well plates (150 000 cells/well), 300 µl volume. Oligonucleotide gymnosis was performed as described for the previous cells.

Live cells imaging of short oligonucleotides accumulation. K562 cells were grown in 96 well plates to 70% confluency and incubated 24h either with 1 µM FAM labelled ORN3 or 1 µM FAM labelled ORN7. After incubation the cells were washed 3 times with PBS to eliminate the labelled oligonucleotides from the media. Image acquisition was done with a DMI600 wide field fluorescence microscope (Leica).

Confocal microscopy. K562 cells (15000 cells/chamber) were grown in a 48 wells/plate in 500 µl of complete Iscove's Modified Dulbecco's Medium. After 36 h of incubation at 5% CO_2 at 37 °C, the cell culture medium was removed and cells rinsed 3 times in PBS and fixed for 10 min with 4% paraformaldehyde in PBS on a 4-chambered 1.0 borosilicate cover glass slide (Labtek), rinsed 3 times with PBS. Permeabilization was then obtained incubating cells in 400 µl of 0.1% Triton X-100 in PBS at room temperature for 15 min, followed by 3 washes with PBS and incubation for 1 hour with blocking buffer (2% BSA in PBS with 0.01% Triton X-100). After 3x5 min washes, cells were incubated with primary antibody diluted in blocking buffer (without Triton X-100) overnight at 4 °C. After 3x5 min washes, cells were incubated with secondary antibodies for 2 h, washed 5min, further stained with nuclear dye Hoechst, (Invitrogen, 1:20000) for 25 min and then washed 3x5 min. Images were acquired using an Olympus confocal FV 3000. Primary antibodies: anti-LIN28A (Cell Signaling 3978,1:250 or Cell Signaling 8641, 1:250). Conjugated antibodies (1:200): Donkey Anti-Rabbit IgG H&L (Alexa Fluor® 594, Abcam, ab150064).

The confocal colocalization images with FAM-labelled RNA-PROTAC were obtained incubating K562 cells (15000 cells/chamber) in a 48 wells/plate with 1 µM FAM-labelled ORN3P_1. After 24 h of incubation at 5% CO_2 at 37 °C, the cell culture medium was removed and cells rinsed 4 times in PBS. The previously described protocol was then follow for the confocal samples preparation.

Immunoblotting. The proteins were extracted using the RIPA buffer (Life technologies) according to the manufacturer's instructions. Protein concentration was assessed with the Pierce BCA protein assay kit (ThermoFisher Scientific).

The protein extracts (12-17 µg) were run on a 4-20% SDS-PAGE and transferred on a nitrocellulose blotting membrane 0.22 µm (GE Healthcare) using the Trans-Blot transfer device (Biorad). Membranes were blocked with 5% milk and incubated with the primary antibody overnight at 4 °C. Membranes were washed with 0.05% PBS-T and reacted with the secondary antibody for 1 h in 5% milk. The proteins were detected using the anti-lin28a antibody (Cell Signaling technology, D9F5), anti-lin28b antibody (Cell Signaling technology, 4196S), anti-rbfox1 (NovusBio, NBP2-13169SS), at 1:1000 and anti-hrnp c antibody (Abcam, AB133607) at 1:10000 dilutions. The western blots were revealed using the Clarity Western ECL substrate (Biorad) on a ChemiDoc MP imager (Biorad). The intensity of the signal was quantified using the ImageJ-Fiji software. Graphical output and statistical analysis (Anova) were generated with GraphPad Prism 7.0 software.

Ubiquitination assay. 22Rv1 cells were seeded into 6 wells/plates and allowed to adhere overnight. On the following day, cells were transfected with 0.745 µg pCMV-myc-Lin28A or 1.5 µg pDEST-Myc-QKI and 1 µg pRK5-HA-Ubiquitin-WT (Addgene plasmid #17608) in Opti-MEM media using Lipofectamine 2000 (Invitrogen). After overnight incubation, Opti-MEM media were replaced with growing media. Cells were treated with indicated concentrations of either vehicle (Opti-MEM) or PROTACs for 48 h at 37 °C. Cells were then placed on ice, rinsed twice with ice-cold 1X PBS and lysed in 500 µL modified 1X RIPA buffer (25 mM Tris-HCl pH 7.6, 150 mM NaCl, 1% NP-40, 1% sodium deoxycholate, 0.1% SDS) containing 1X protease inhibitor cocktail (Roche), 10mM iodoacetamide (MW=185, 92.5mg in 5ml, 10x) and 20 µM PR-619 (MW=223.3, 2.23mg in 5ml, 100x). Protein lysate was normalized and 300 µg of lysate was aliquoted onto 40 µL (bed volume) of Dynabeads™ Protein G (Catalog Nos. 10003D, 10004D, 10009D). cMyc containing proteins were immunoprecipitated from cells lysates overnight at 4 °C with gentle rotation, after which samples were spun down at 3000 × g at 4 °C for 2 min and the beads were washed once with ice-cold lysis buffer and three times with ice-cold 1X PBS-T. Beads were resuspended in 30ul SDS sample buffer (Laemmli) containing 5% 2-mercaptoethanol (BME). Immunoprecipitated proteins were eluted off of the beads by heating at 95 °C for 8 min and the supernatant was run on an SDS-PAGE gel and evaluated for the presence of immunoprecipitated cMyc-tagged proteins, as well as ubiquitinated HA-tagged proteins (anti-HA-tag (6E2), CST #2367).

ELISA Assay. K562 cells were plated at 480000 cells/well in a 24 wells/plate, in 0.3 mL fresh medium (IMDE + 10 % FBS + 4 mM Q) at 37°C, 5% CO_2. For gymnosis experiments, K562 cells were plated in 24 wells/plates (480000 cells/well), 325 µl volume. Oligonucleotide gymnosis was performed adding to the cells 25 µl of Oligo PROTAC in Opti-MEM (31985-047, Gibco) at 2 µM. After 24 h, the supernatants were removed and lysates of PBS-washed K562 cells were obtained by 3h incubation on ice in 40 µl of lysate buffer of 50mM HEPES, pH 7.5, 150 mM KCl, 1% NP40, 0.5 mM DTT, 0.1% SDS, 10nM $ZnCl_2$, complete protease inhibitor EDTA-free (Roche) followed by several pipetting in order to disrupt the cells. Before analysis the lysate was diluted in extra 400 µl of lysate buffer. Streptavidin coated white Pierce plates (Thermofisher, Cat. no. 15502) were blocked with Tris-buffered saline (25 mM Tris, 150 mM NaCl; pH 7.2), 0.1% BSA, 0.05% Tween for 20 min at 4 °C. As previously described,[1] lysates stored for prolonged periods even at 80 °C showed reduced activity, particularly for Lin28, therefore the experiments were all run in a 4 days range of time and all incubation and washing before the fixation were done in cold buffers and at low temperatures.

Sample type A (gymnosis): the lysate obtained by K562 pre-incubated with biotinylated PROTAC was allowed to bind to the plate for 3 h at 4 °C. The wells were then washed with cell lysate buffer, emptied and exposed to a formaldehyde solution [0.5% in phosphate buffered saline (PBS)] for 5 min. This step was intended to prevent dissociation of the RNA from the RBP during subsequent steps. In our experiments, the fixation step stabilized the interaction and was retained in all of our microtiter plate experiments.

Sample type B (lysate): 3'-biotinilated RNA-PROTACs were diluted to 100nM in cell lysate buffer and allowed to bind for 1 h at 4 °C. The wells were not washed and cell lysates added for 2h at 4°C. The wells were then washed with cell lysate buffer, emptied and exposed to a formaldehyde solution [0.5% in phosphate buffered saline (PBS)] for 5 min.

Sample type C (lysate pre-incubated): 3'-biotinylated ORN3P1/ORN7P1 were pre-incubated with K562 lysate for 2 h on ice and then allowed to bind to the plate for 1 h at 4°C.

The bound proteins of interest were detected with using the anti-lin28a antibody (Cell Signaling technology, D9F5), at 1:1500, anti-tls/fus antibody (Abcam, AB23439) at 1:500 dilutions. The buffer for primary and secondary peroxidase conjugated antibodies was 25 mM Tris, 150 mM NaCl; pH 7.2), 0.1% BSA, 0.05% Tween. The peroxidase conjugates against rabbit IgG (074-1506, BioConcept AG, Allschwil, Switzerland) were applied at a dilution of 1:2000 for 30 min. The BM chemiluminescenCE ELISA substrate (POD) was used for signal generation.

Fluorescence-activated flow cytometry (FACS). K562 cells were incubated 48h with compounds ORN3P_1, ORN7P_1, ORN4P_1 and correspondent controls ORN3P_2, ORN7P_2, ORN4P_2 at 2 µM and 0.5 µM concentrations, pelleted, washed with phosphate-buffered saline (PBS), suspended in 200 µl FACS buffer (PBS + 2% fetal bovine serum), and acquired on a BD LSRFortessa analyzer (BD Biosciences).

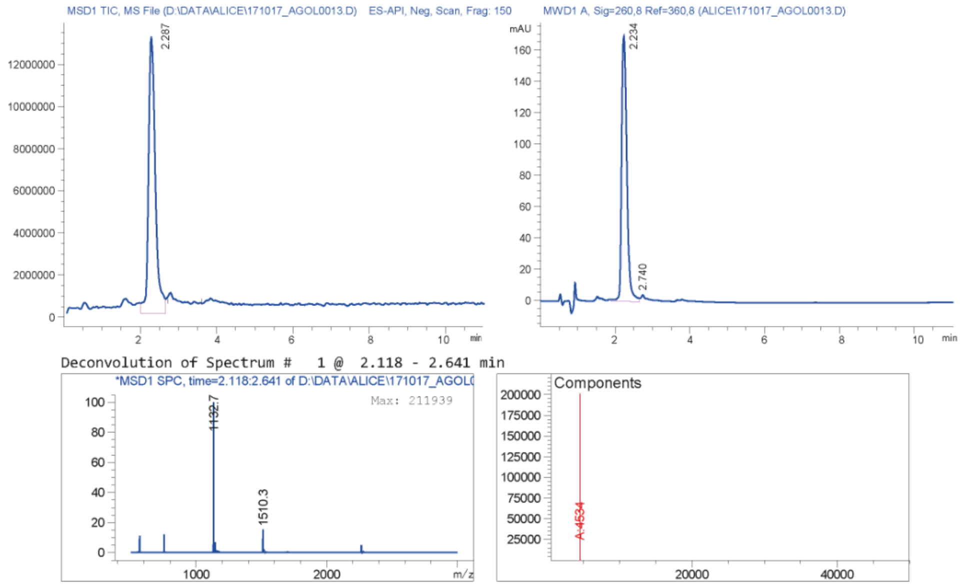

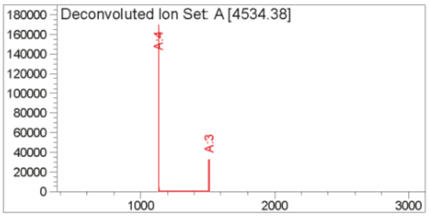

Component	Molecular Weight	Absolute Abundance	Relative Abundance
A	4534.38	201093	100.00

ORN1

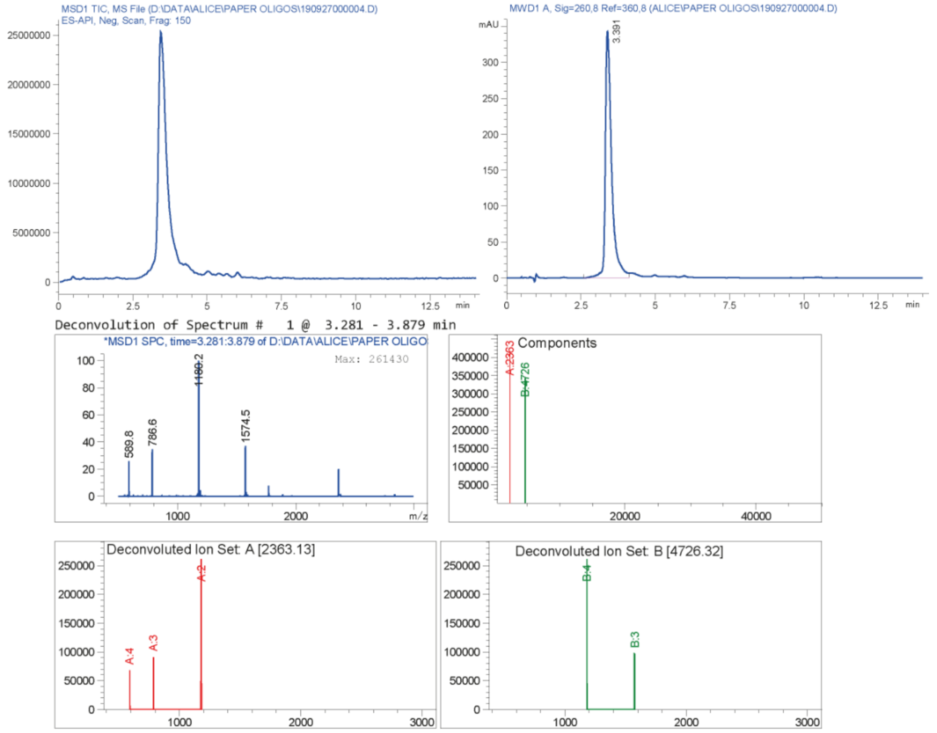

Component	Molecular Weight	Absolute Abundance	Relative Abundance
A	2363.13	412541	100.00
B	4726.32	342479	83.02

ORN2

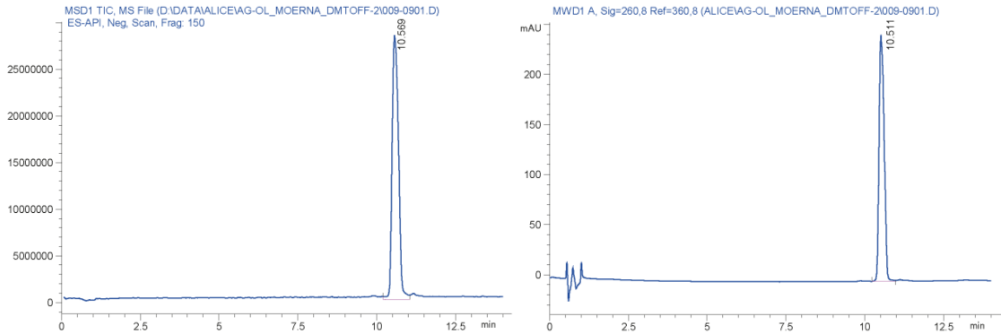

Deconvolution of Spectrum # 1 @ 10.338 - 10.836 min

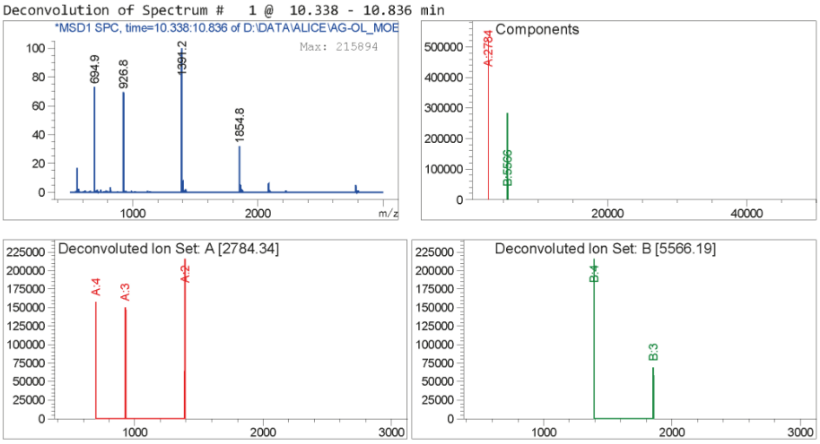

Component	Molecular Weight	Absolute Abundance	Relative Abundance
A	2784.34	523552	100.00
B	5566.19	284398	54.32

ORN3

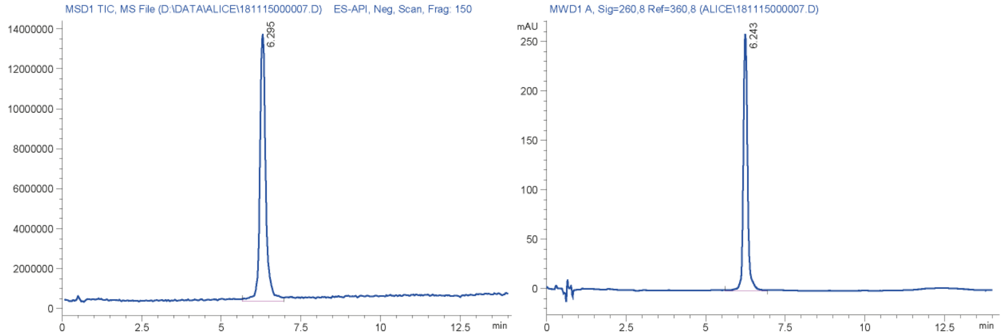

Deconvolution of Spectrum # 1 @ 6.153 - 6.527 min

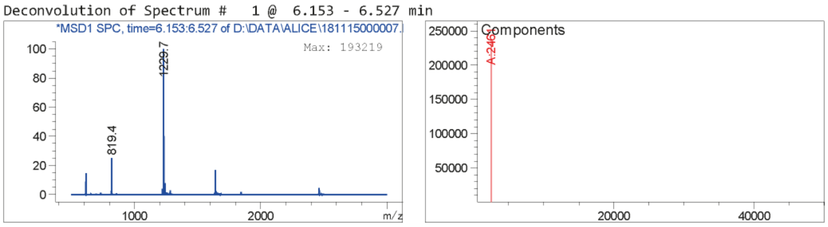

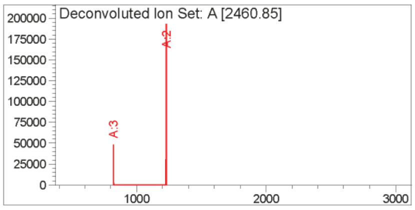

Component	Molecular Weight	Absolute Abundance	Relative Abundance
A	2460.85	235886	100.00

ORN4

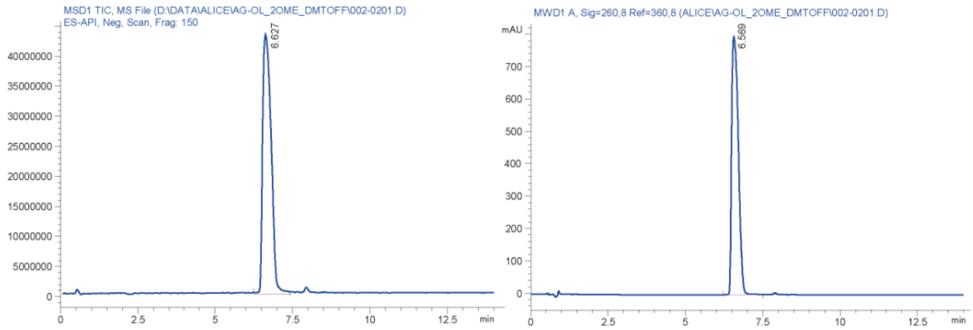

Deconvolution of Spectrum # 1 @ 6.328 - 7.050 min

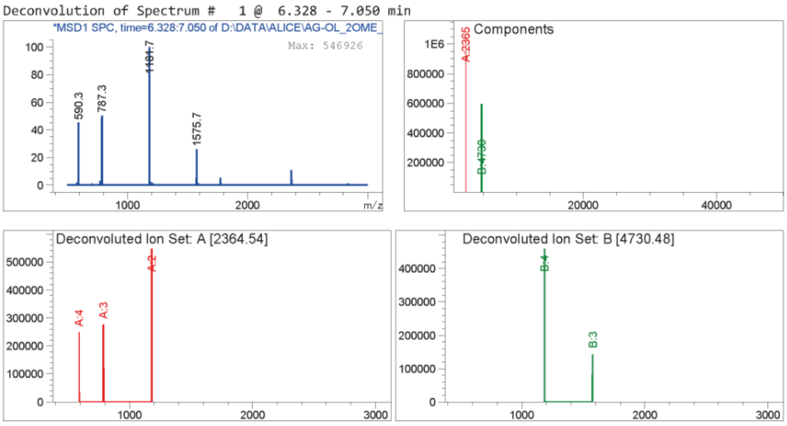

Component	Molecular Weight	Absolute Abundance	Relative Abundance
A	2364.54	1033591	100.00
B	4730.48	594909	57.56

ORN6

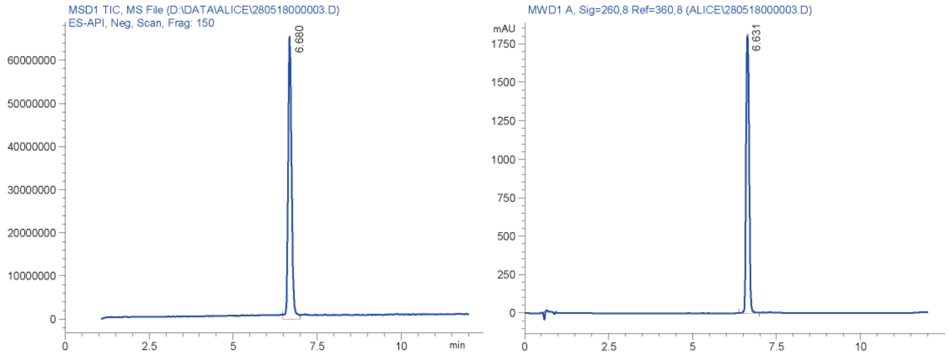

Deconvolution of Spectrum # 1 @ 6.529 - 6.903 min

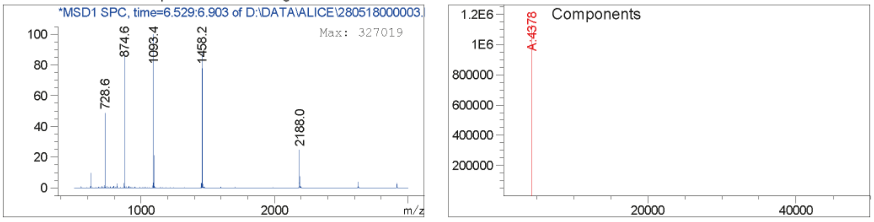

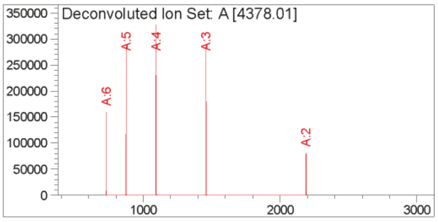

Component	Molecular Weight	Absolute Abundance	Relative Abundance
A	4378.01	1127587	100.00

ORN7

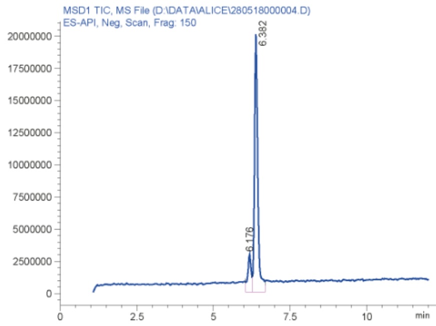

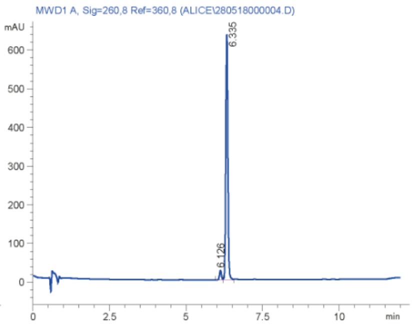

Deconvolution of Spectrum # 1 @ 6.280 - 6.529 min

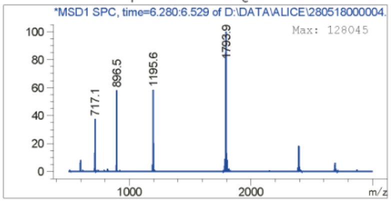

Signal 2: MWD1 A, Sig=260,8 Ref=360,8

Peak #	RetTime [min]	Type	Width [min]	Area [mAU*s]	Height [mAU]	Area %
1	6.126	VV	0.0725	110.72843	24.60912	3.5600
2	6.335	VV	0.0731	2999.63745	635.14124	96.4400
Totals :				3110.36588	659.75035	

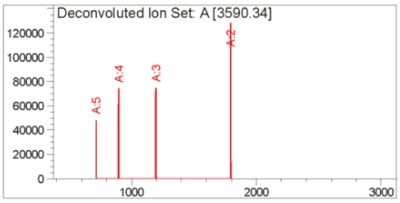

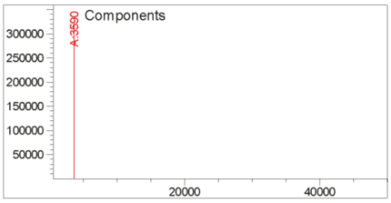

Component	Molecular Weight	Absolute Abundance	Relative Abundance
A	3590.34	321256	100.00

ORN8

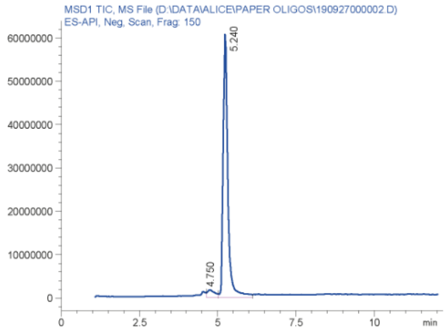

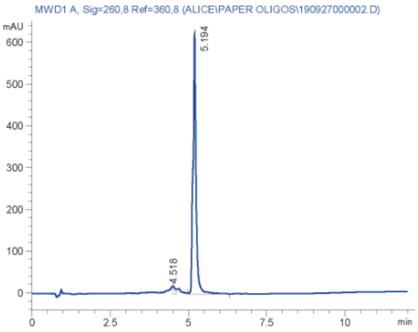

Deconvolution of Spectrum # 1 @ 5.110 - 5.459 min

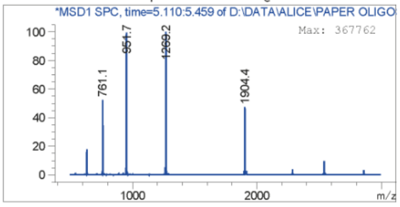

Signal 2: MWD1 A, Sig=260,8 Ref=360,8

Peak #	RetTime [min]	Type	Width [min]	Area [mAU*s]	Height [mAU]	Area %
1	4.518	VV	0.0704	94.74182	18.31457	2.1856
2	5.194	VV	0.0930	4240.13379	626.12048	97.8144
Totals :				4334.87561	644.43505	

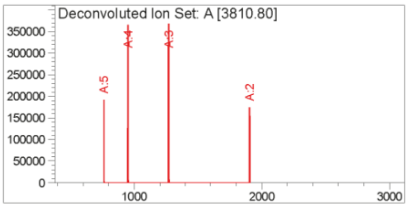

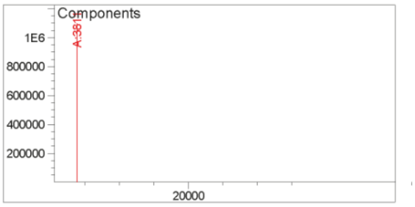

Component	Molecular Weight	Absolute Abundance	Relative Abundance
A	3810.80	1097323	100.00

ORN3P$_1$

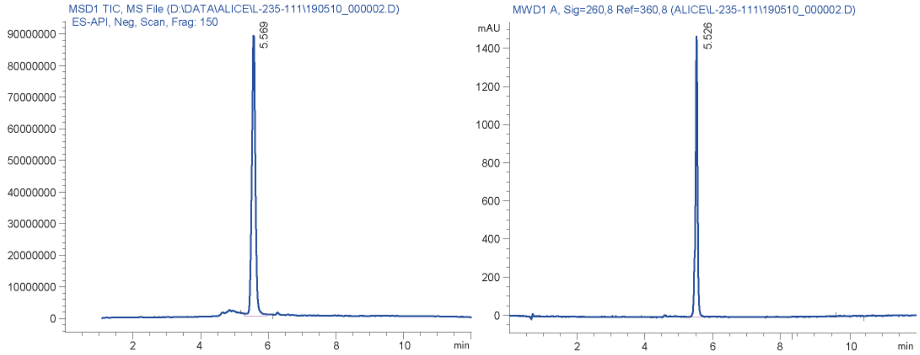

Deconvolution of Spectrum # 1 @ 5.358 - 5.757 min

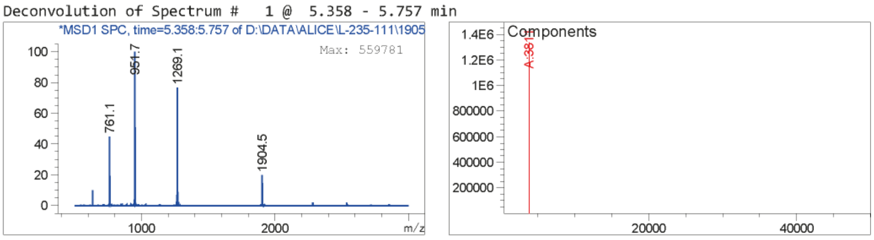

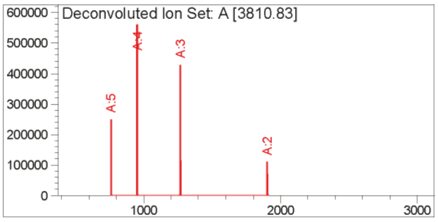

Component	Molecular Weight	Absolute Abundance	Relative Abundance
A	3810.83	1335134	100.00

ORN3P$_{con}$

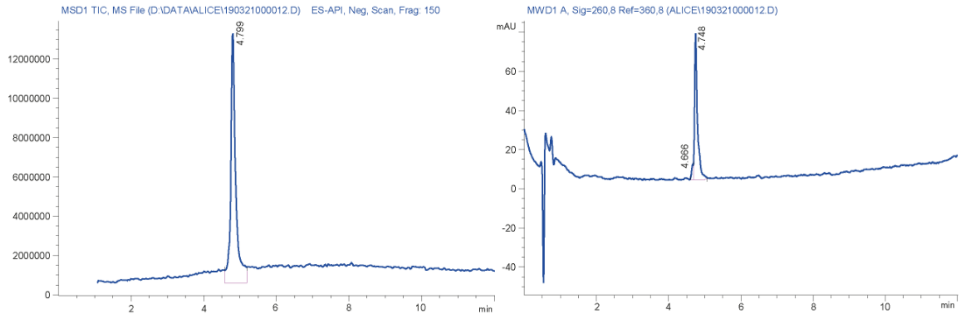

Deconvolution of Spectrum # 1 @ 4.661 - 5.010 min

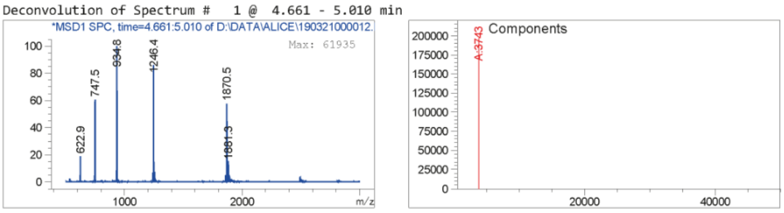

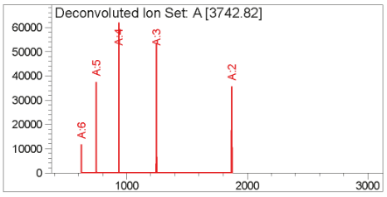

Component	Molecular Weight	Absolute Abundance	Relative Abundance
A	3742.82	196637	100.00

ORN5P$_1$

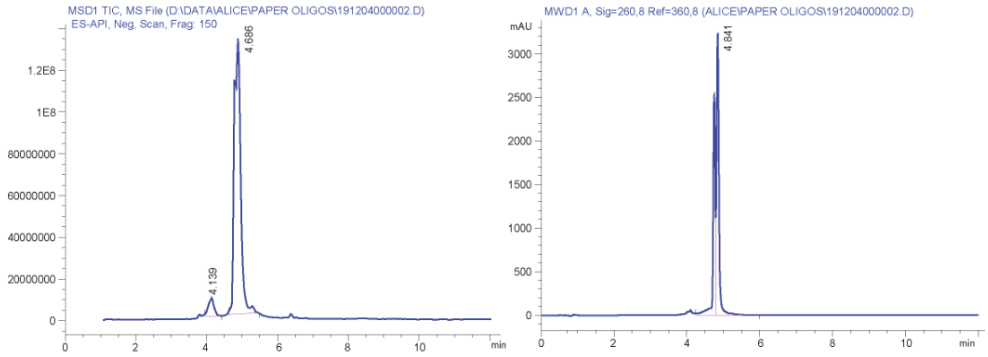

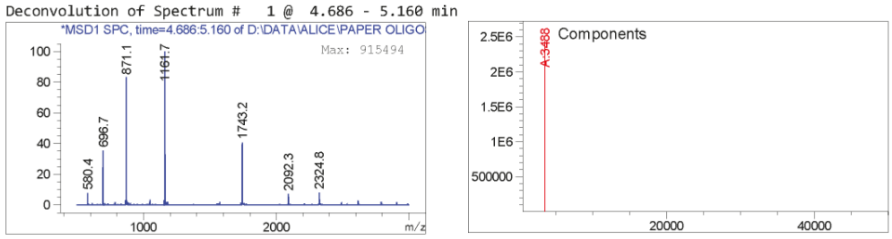

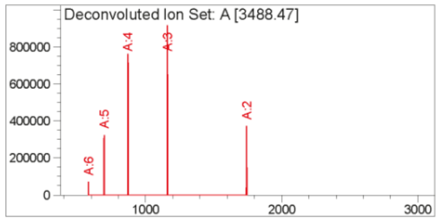

Component	Molecular Weight	Absolute Abundance	Relative Abundance
A	3488.47	2434916	100.00

ORN4P$_1$

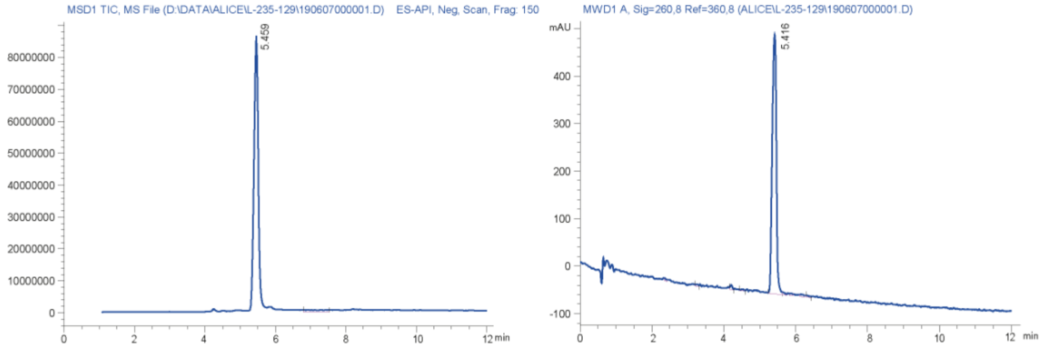

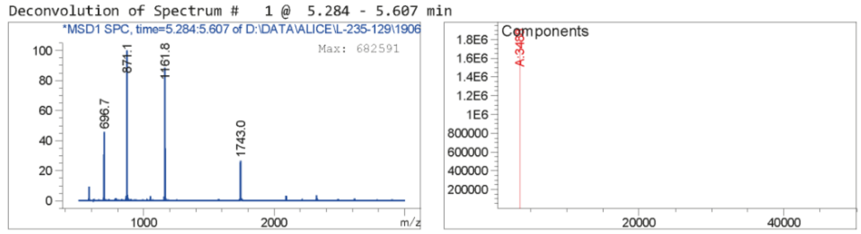

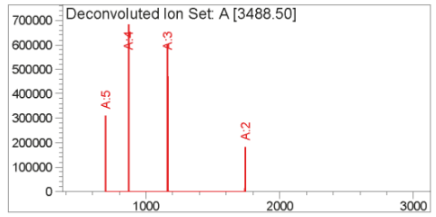

Component	Molecular Weight	Absolute Abundance	Relative Abundance
A	3488.50	1775072	100.00

ORN4P$_{con}$

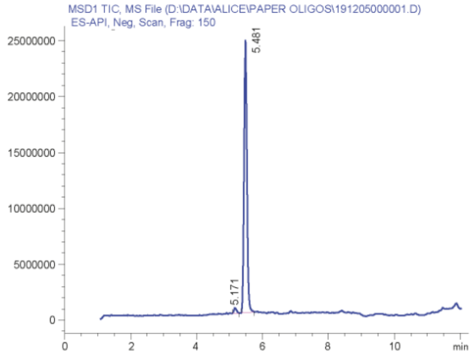

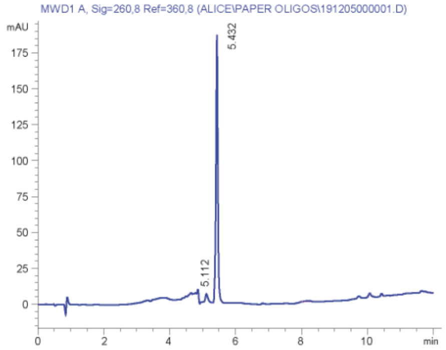

Deconvolution of Spectrum # 1 @ 5.558 min

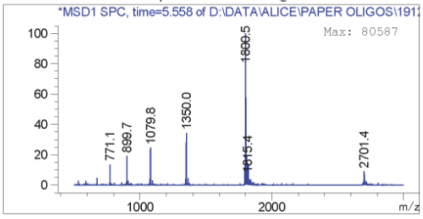

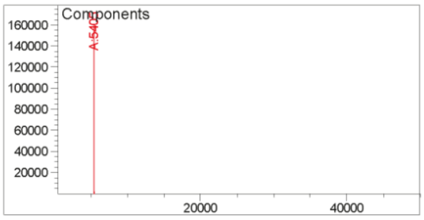

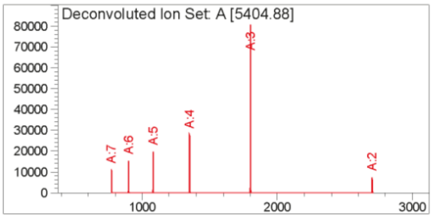

Component	Molecular Weight	Absolute Abundance	Relative Abundance
A	5404.88	159702	100.00

ORN7P$_1$

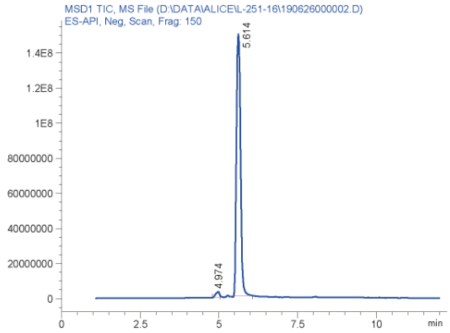

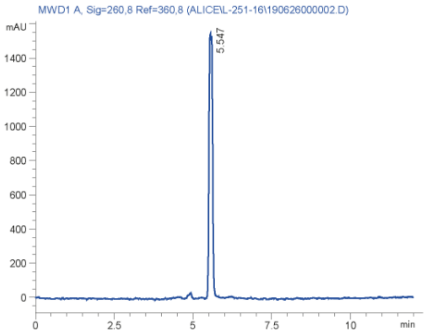

Deconvolution of Spectrum # 1 @ 5.483 - 5.881 min

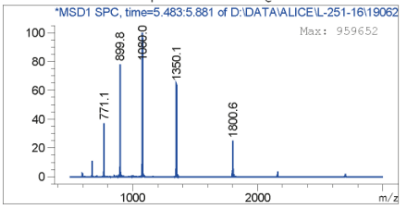

Signal 2: MWD1 A, Sig=260,8 Ref=360,8

Peak #	RetTime [min]	Type	Width [min]	Area [mAU*s]	Height [mAU]	Area %
1	5.547	VV	0.0594	6306.89258	1552.86108	52.8251
2	5.591	VV	0.0607	5632.29834	1545.82483	47.1749
Totals :				1.19392e4	3098.68591	

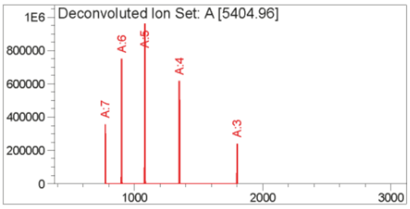

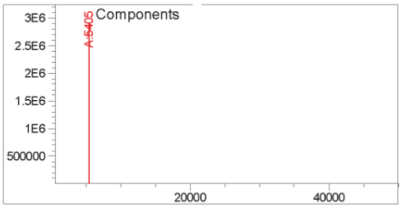

Component	Molecular Weight	Absolute Abundance	Relative Abundance
A	5404.96	2894381	100.00

ORN7P$_{con}$

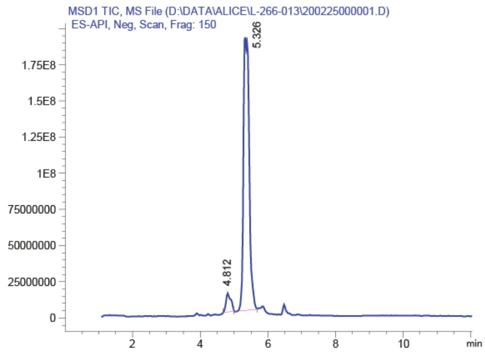

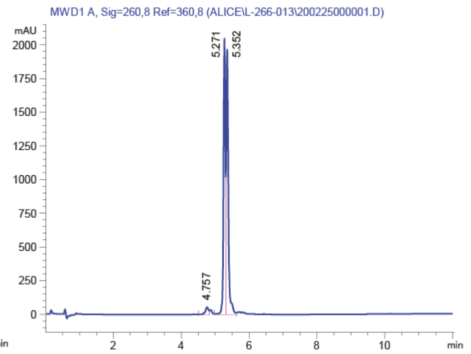

Deconvolution of Spectrum # 1 @ 5.160 - 5.608 min

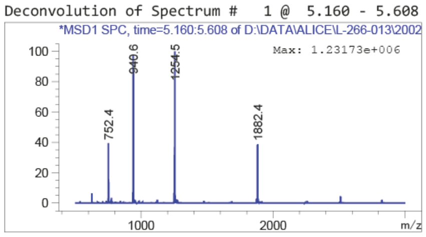

Peak #	RetTime [min]	Type	Width [min]	Area [mAU*s]	Height [mAU]	Area %
1	4.757	VV	0.1037	396.30972	55.23082	2.3697
2	5.271	VV	0.0583	7806.33594	2057.10840	46.6764
3	5.352	VV	0.0644	8521.72656	1972.68506	50.9539

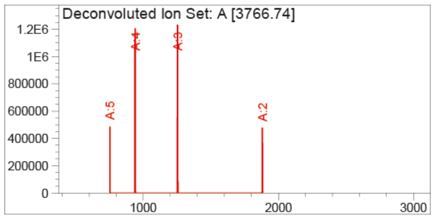

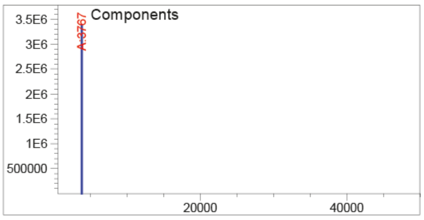

Component	Molecular Weight	Absolute Abundance	Relative Abundance
A	3766.74	3381050	100.00

*** End of Report ***

ORN9P1

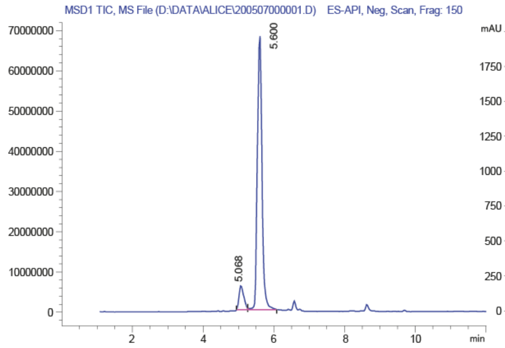

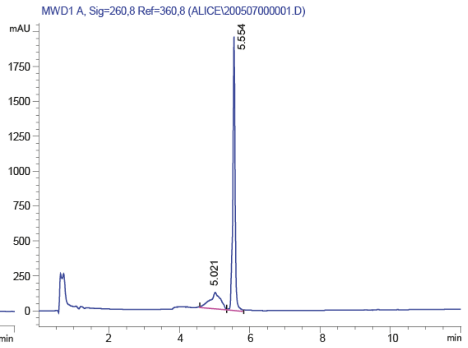

Deconvolution of Spectrum # 1 @ 5.459 - 5.807 min

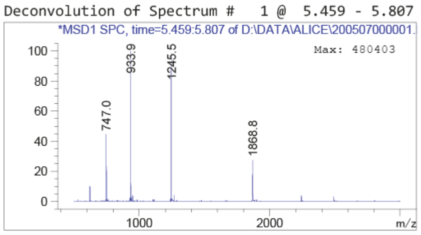

Peak #	RetTime [min]	Type	Width [min]	Area	Height	Area %
1	4.934	MF	0.1757	1.07273e8	1.01744e7	3.9287
2	5.410	FM	0.2010	2.62322e9	2.17504e8	96.0713

Totals : 2.73049e9 2.27679e8

Component	Molecular Weight	Absolute Abundance	Relative Abundance
A	3739.92	1298208	100.00

*** End of Report ***

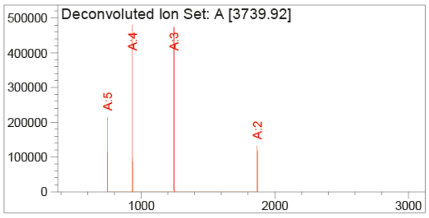

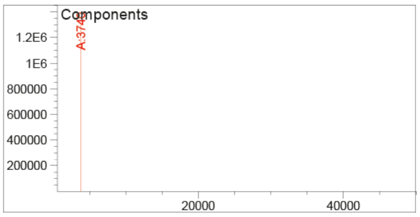

ORN10P1

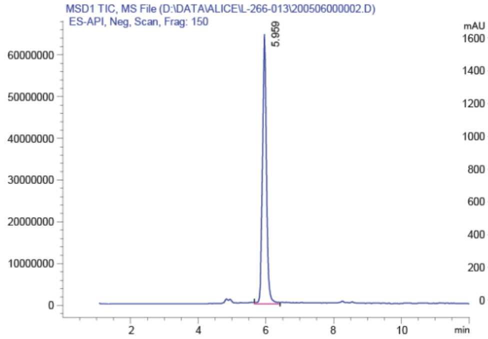

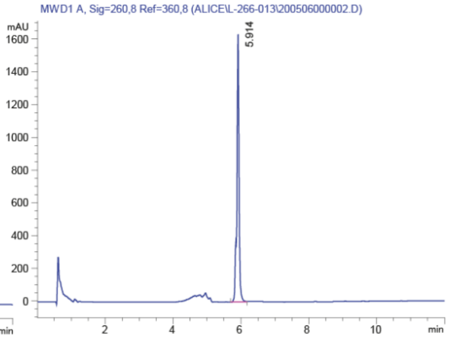

Deconvolution of Spectrum # 1 @ 5.807 - 6.131 min

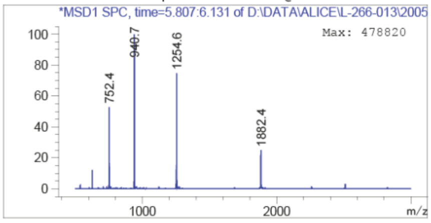

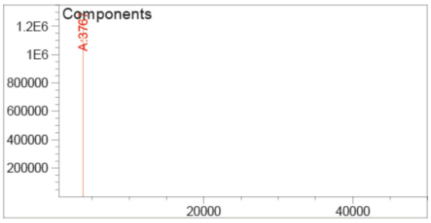

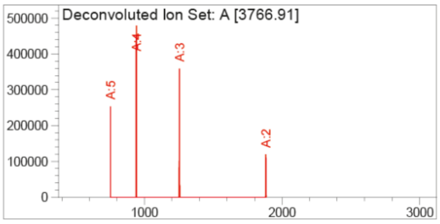

Component	Molecular Weight	Absolute Abundance	Relative Abundance
A	3766.91	1207754	100.00

*** End of Report ***

ORN9P$_{con}$

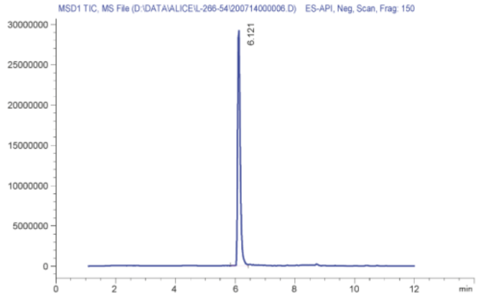

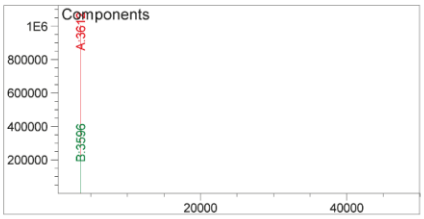

Deconvolution of Spectrum # 1 @ 6.081 - 6.156 min

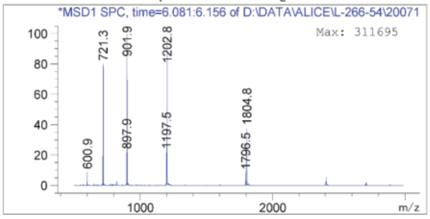

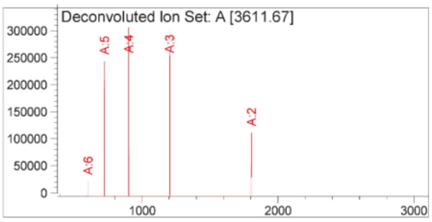

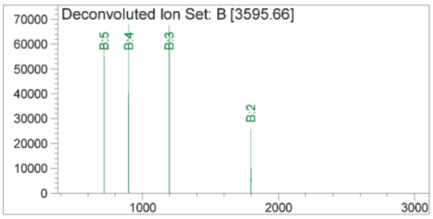

Component	Molecular Weight	Absolute Abundance	Relative Abundance
A	3611.67	1004911	100.00
B	3595.66	220427	21.93

ORN3VH$_{032}$

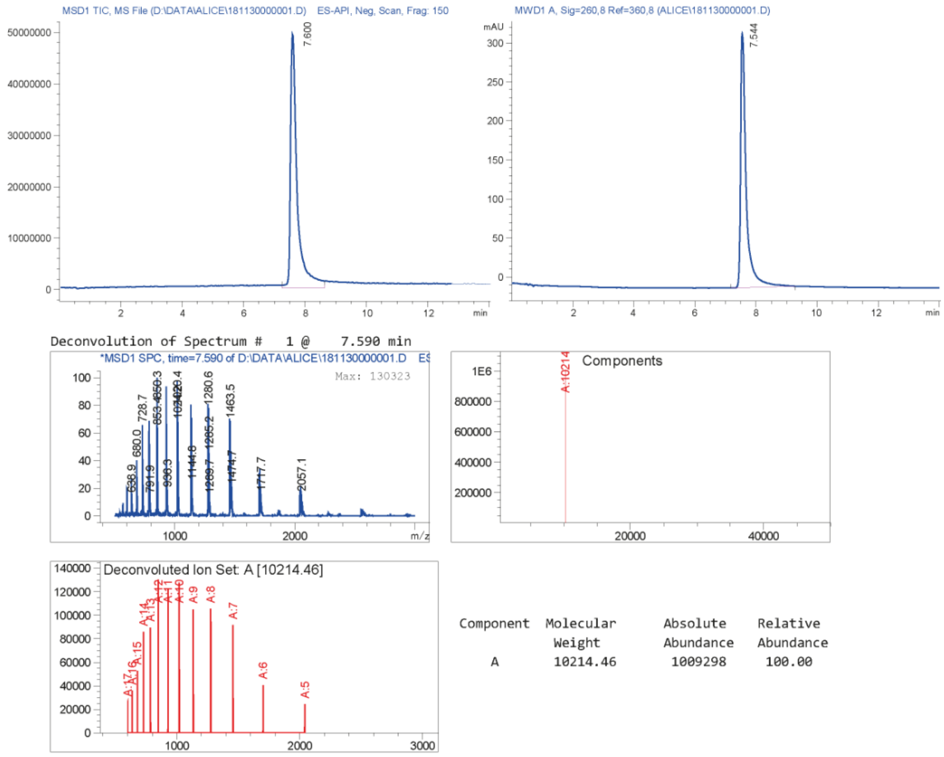

PreE-let-7-f1_FAM

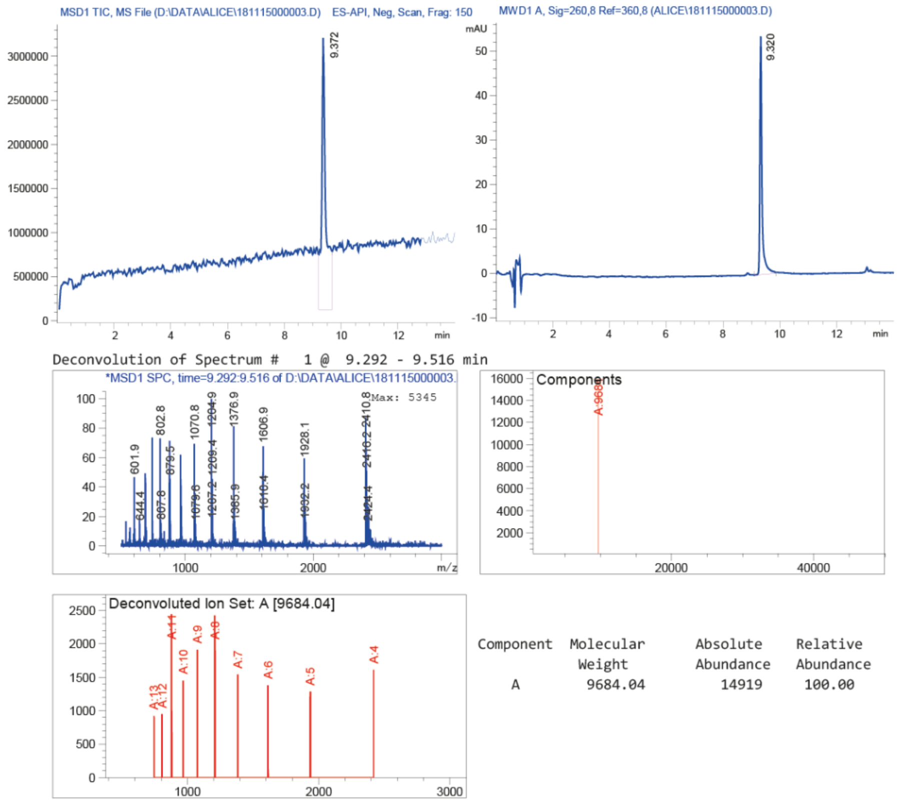

PreE-let-7-f1

Figure S1. LCMS chromatograms of oligonucleotides and RNA-PROTACS. Reverse-phase high performance liquid chromatography mass spectrometry (LCMS) chromatograms of oligonucleotide sequences and oligonucleotide PROTACs used in this study.

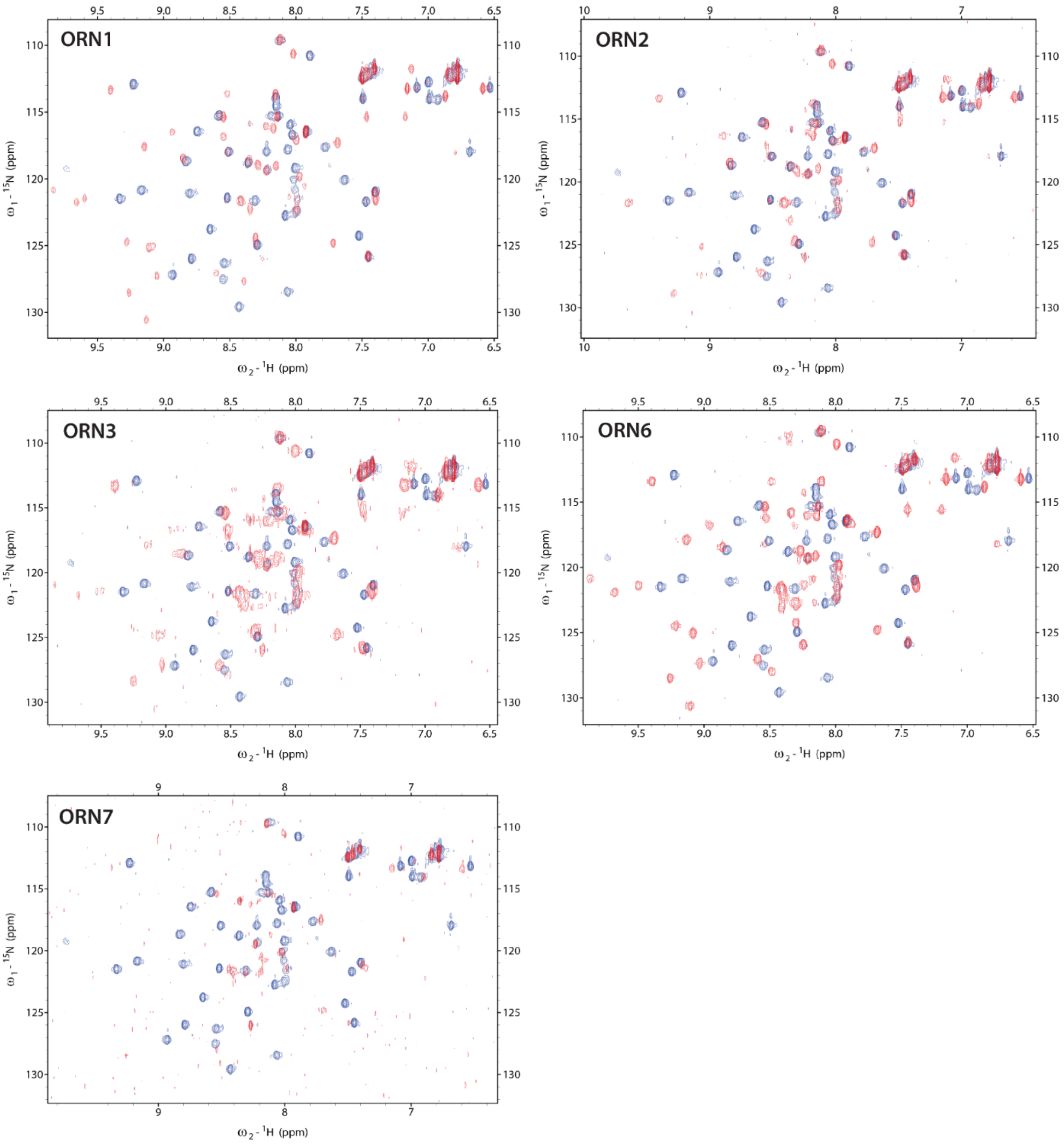

Figure S2. ^{1}H-^{15}N HSQC spectra of Lin28_ZKD and oligonucleotides.
Overlay of ^{15}N HSQC spectra of Lin28_ZKD, free (blue) and bound (red) to **ORN1**, **ORN2**, **ORN3**, **ORN6**, **ORN7**. The complexes between oligonucleotides and Lin28_ZKD were formed in 10 mM HEPES pH 7.0 by adding ssRNA AGGAGAU or the listed modified oligo into Lin28ZKD at 1:1 stoichiometry and monitoring at 283K.

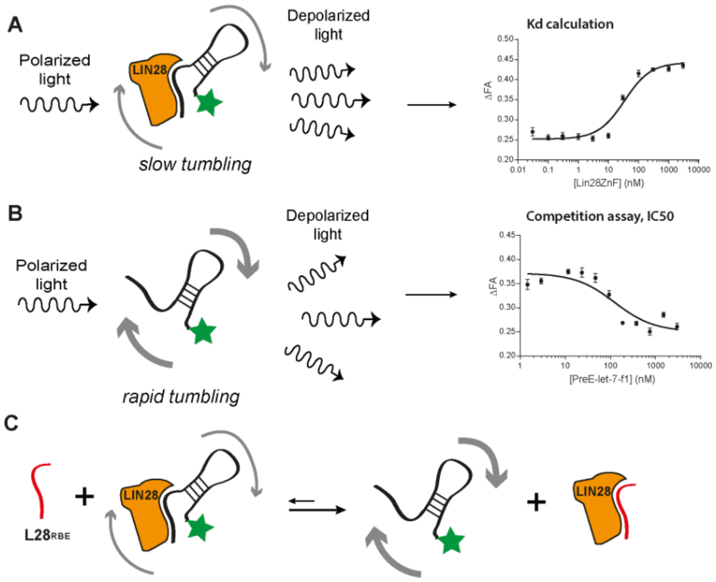

Figure S3. Lin28 fluorescence competition assay.
In the fluorescence polarization assay, LIN28_ZKD was titrated into solutions containing 2 nM FAM-labeled preE-let-7f-1 probes and after incubation, the fluorescence polarization has been measured. For FP competition assay the binding ability of FAM-labelled preE-let-7f-1 is still measured but in the presence of an unlabeled competitor. A decrease in FP occurs because unlabeled oligonucleotides compete with the FAM-labelled probe for binding Lin28_ZKD. To setup the assay, a fixed concentration of Lin28_ZKD (40 nM) along with 10 nM FAM-labeled preE-let-7f-1 was added into a 384-well plate. Serial diluted oligonucleotide sequences where then added to the plate. The resulting mixture was equilibrated for 30 min at 25 °C prior to measuring FP. The data was fit using GraphPad Prism 7.0.

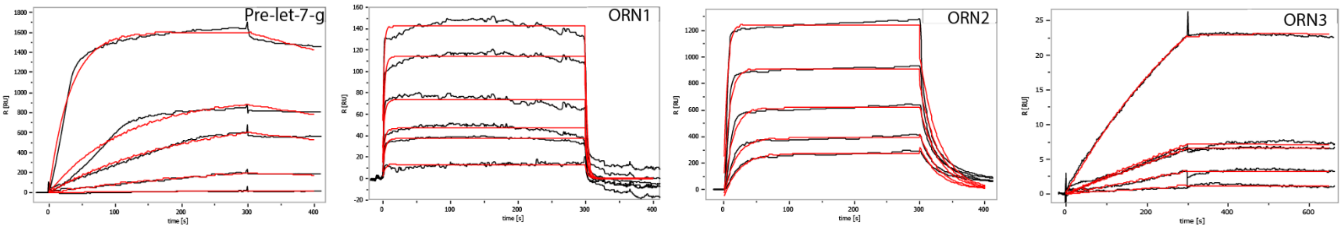

	Kd (µM)	St Dev	k_{on} [1/M*s]	k_{off} [1/s]
**Pre-let-7-g**	0.0087	±0.0016	1.30E+05	1.13E-03
**ORN1**	25.8	±17.6	1.73E+04	3.56E-01
**ORN2**	0.55	±0.10	5.39E+04	2.83E-02
**ORN3**	0.58	±0.20	1.65E+03	1.16E-03

Figure S4. Surface Plasma Resonance of Lin28_ZKD and oligonucleotides.
In black, sensor-grams of modified oligoribonucleotides **ORN1**, **ORN2**, **ORN3**, **ORN7** and **ORN8** binding to immobilized LIN28_ZKD. Sensorgrams show pairs of duplicate runs measured at 5-6 graded concentrations; red lines correspond to curve fittings for each concentration. Binding experiments were performed at 285.15 K in SICK kinetic format. For each sensor-gram, values shown represent fitted dissociation constants for complex formation.

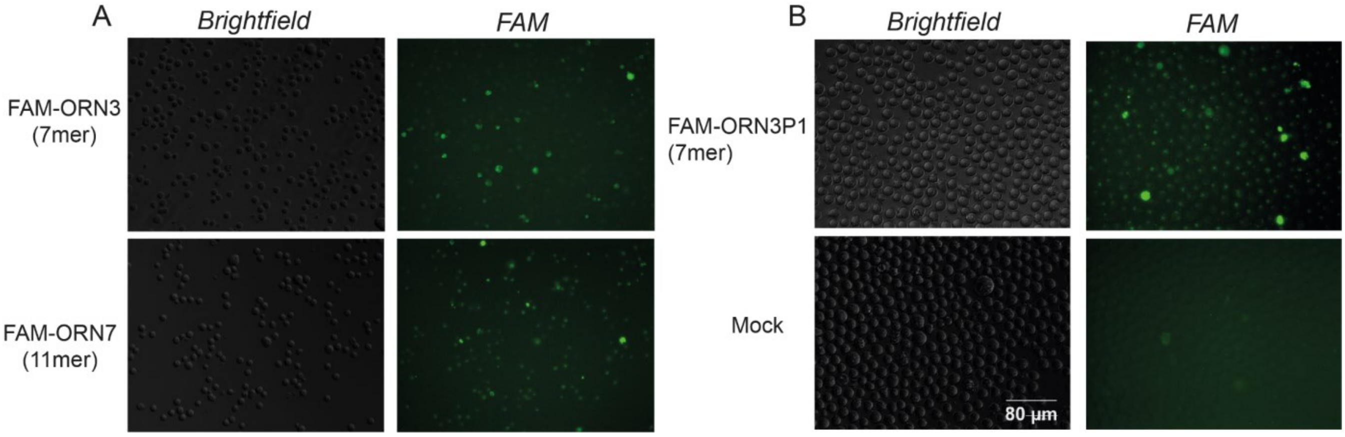

Figure S5. Intracellular uptake of FAM-labelled oligonucleotides and RNA-PROTAC ORN3P$_1$.
Fluorescence microscopy images generated after 24 h incubation of 1 µM FAM labelled A) **ORN3** and **ORN7** or B) **ORN3P$_1$**. ORN3P1 is present in the cytoplasm and nuclei of K562 cells.

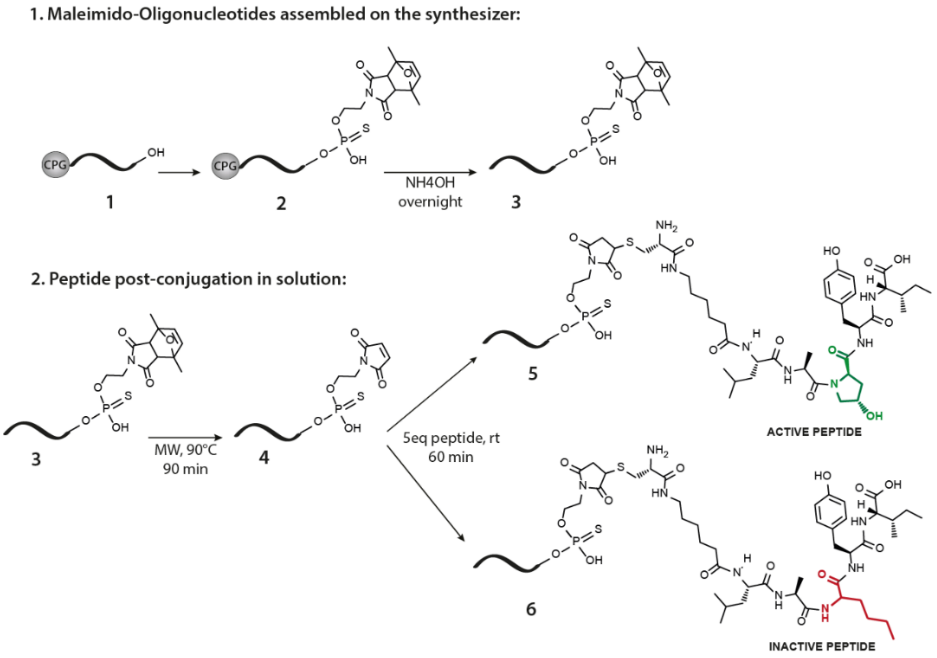

Figure S6. Solid-phase synthesis of RNA-PROTACs and their controls.
The 5'-maleimide modifier was prepared at 0.1 M concentration and the synthesis of the oligonucleotides PROTAC sequences (**2**) was conducted following standard parameters. The CPG was then suspended overnight at 35°C in 25 % ammonia (**3**). Purification protocol was done as described for the oligonucleotide sequences. Retro-Diels Alder reaction released the masked maleimide to obtain product **4**: 100 µM solution of the 5'-maleimide intermediate (**3**) in water was microwave-irradiated for 90 min at 90 °C, followed by partial evaporation. A 10 mg/ml aqueous solution of peptide (Genscript) was added to the concentrated solution of **4** (5eq). The reaction was buffered with TEAA at pH 7 and allowed to run for 1 h at RT. Product **5** and **6** were purified by RP-HPLC as described in Materials and methods section.

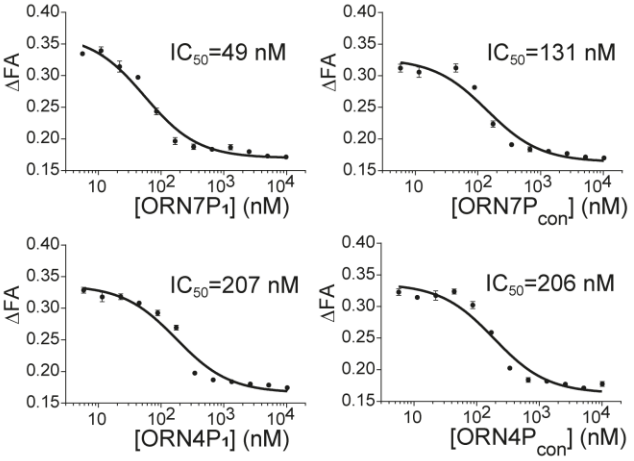

Name	Sequence	Peptide[a]	*IC*50 (nM)
**ORN3P$_1$**	AGGAGAU	C[Ahx]LA[Hyp]YI	101 ± 67.9
**ORN5P$_1$**	UUUAUUG	C[Ahx]LA[Hyp]YI	623.4 ± 330.6
**ORN3P$_{con}$**	AGGAGAU	C[Ahx]LA[Nle]YI	*
**ORN4P$_1$**	AGGAGAU	C[Ahx]LA[Hyp]YI	207.8 ± 27.4
**ORN4P$_{con}$**	AGGAGAU	C[Ahx]LA[Nle]YI	206.0 ± 2.1
**ORN7P$_1$**	AGGAGAUAACU	C[Ahx]LA[Hyp]YI	49.3 ± 22.2
**ORN7P$_{con}$**	AGGAGAUAACU	C[Ahx]LA[Nle]YI	131.2 ± 17.3

* Not determined

Figure S7. Fluorescence polarization assay for RNA-PROTACs ORN3P$_1$, ORN7P$_1$ and ORN4P$_1$.

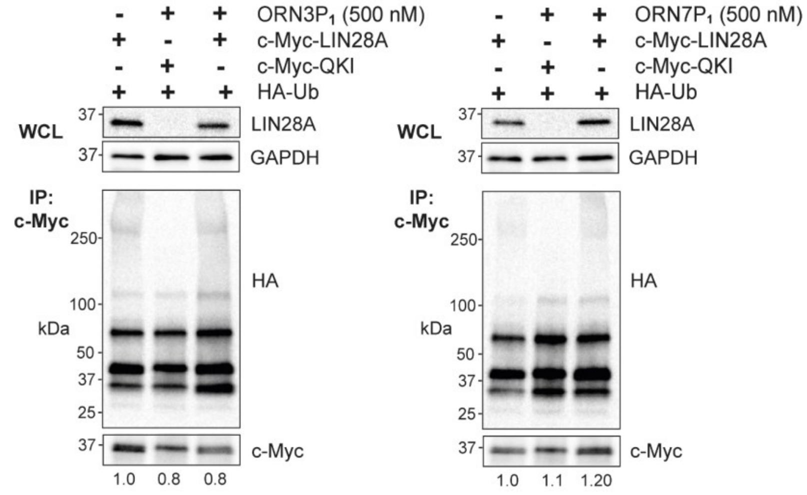

Figure S8. RNA-PROTAC-mediated ubiquitination of myc-Lin28A in 22Rv1 cells (replicate).
RNA-PROTAC-mediated ubiquitination of myc-Lin28A in 22Rv1 cells co-transfected with HA-Ubiquitin (HA-Ub) and pCMV-myc-Lin28A or pDEST-Myc-QKI with either vehicle (OptiMEM) or 500 nM **ORN3P$_1$** or **ORN7P$_1$**. cMyc-immunoprecipitated lysates were separated by SDS-PAGE followed by western blots detecting HA (Ub). Immune-quantification numbers below the membranes corrects for the c-Myc immunoprecipitation step. Representative replicate.

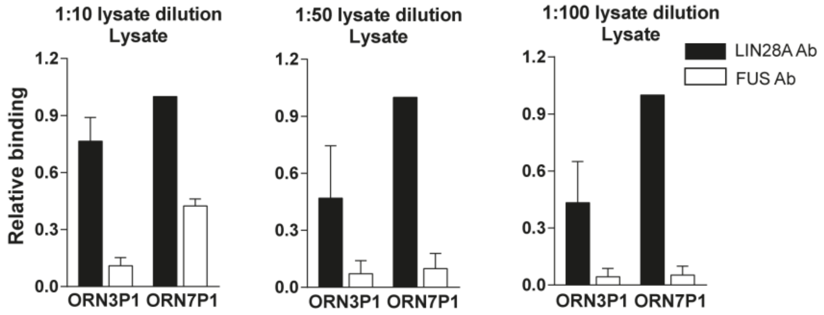

Gymnosis (Abs)

	Lin28A			FUS		
	1/10	*1/50*	*1/100*	*1/10*	*1/50*	*1/100*
**Mock**	436488	230389	301782	2730303	3512587	12937003
**ORN3P1**	862560	540788	591502	446151	263458	408700
**ORN7P1**	741607	771768	1408893	847243	466495	736516
**Mock**	1089703	848797	891657	6063779	6423719	13366765
**ORN3P1**	2413303	1164569	1499985	1926510	1078913	1999458
**ORN7P1**	1317083	1945518	2191796	2866218	1553225	2307586
**Mock**	2361208	2264826	2082215	8882873	9204954	15265015
**ORN3P1**	2600114	1358679	1831692	2803614	1559716	2996388
**ORN7P1**	1544299	2178706	2585834	4308826	2295705	3601261

Lysate incubation (Abs)

	Lin28A			FUS		
	1/10	*1/50*	*1/100*	*1/10*	*1/50*	*1/100*
**Mock**	436488	230389	301782	2730303	3512587	12937003
**ORN3P1**	1369678	2725139	11211957	490338	336034	611536
**ORN7P1**	10409946	8690663	17318668	3021831	818536	1417801
**Mock**	1089703	848797	891657	6063779	6423719	13366765
**ORN3P1**	2554464	1732900	2140182	1662170	1510602	2570299
**ORN7P1**	3129867	3565537	4889519	7002486	2357990	3281663
**Mock**	2361208	2264826	2082215	8882873	9204954	15265015
**ORN3P1**	5378158	4350704	4730952	3740694	3532986	4858495
**ORN7P1**	6285077	5911347	7301605	9539015	4402236	5490757

Figure S9. Pull-down of Lin28A by RNA-PROTACs from K562 cells. For the Lysate incubation experiment, the biotinylated RNA-PROTACs was incubated on the streptavidin coated plate for 1 h at 4 ˚C. The PROTAC bound plates were then incubated with K562 lysate for 2 h. The background is expressed as ratio between the mock value in the assay and the maximal value on the plate. Table with original absorbance values for both the gymnosis and lysate ELISA assay. The table contains the raw data. Data analysis as done in Towbin et al., Nucleic Acids Research 2012, 41.

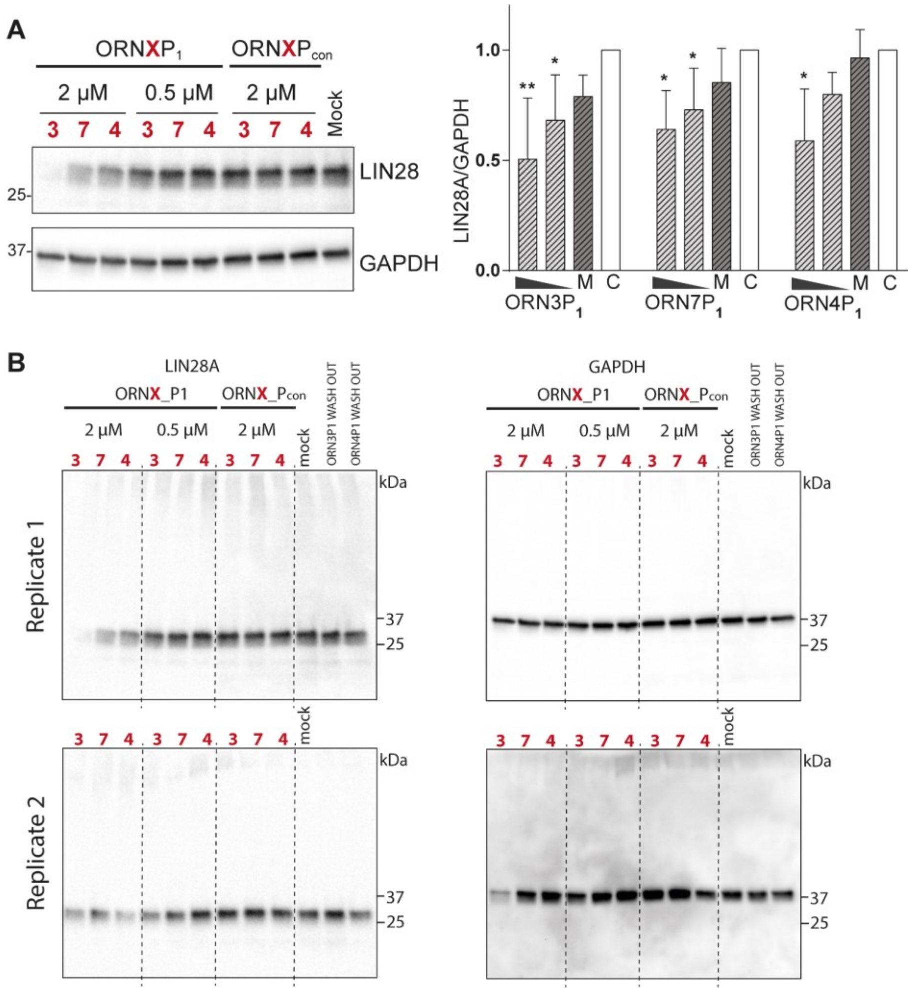

Figure S10. Western blot membranes for Lin28A and GAPDH detection in NT2.D1 cells. A) Western blot and quantification of Lin28A from NT2/D1 cells after treatment with **ORN3P$_1$** and **ORN7P$_1$** and controls. M: cells treated with mock solutions; C: cells treated with correspondent control. B) Uncropped western blots of Lin28A from NT2/D1 cells after treatment with **ORN3P$_1$**, **ORN7P$_1$** and **ORN4P$_1$** and corresponding controls. Protein extracts were analyzed by immunoblotting with the indicated antibody. Data are shown as mean ±SD of three independent experiments. Error bars indicate standard deviations.

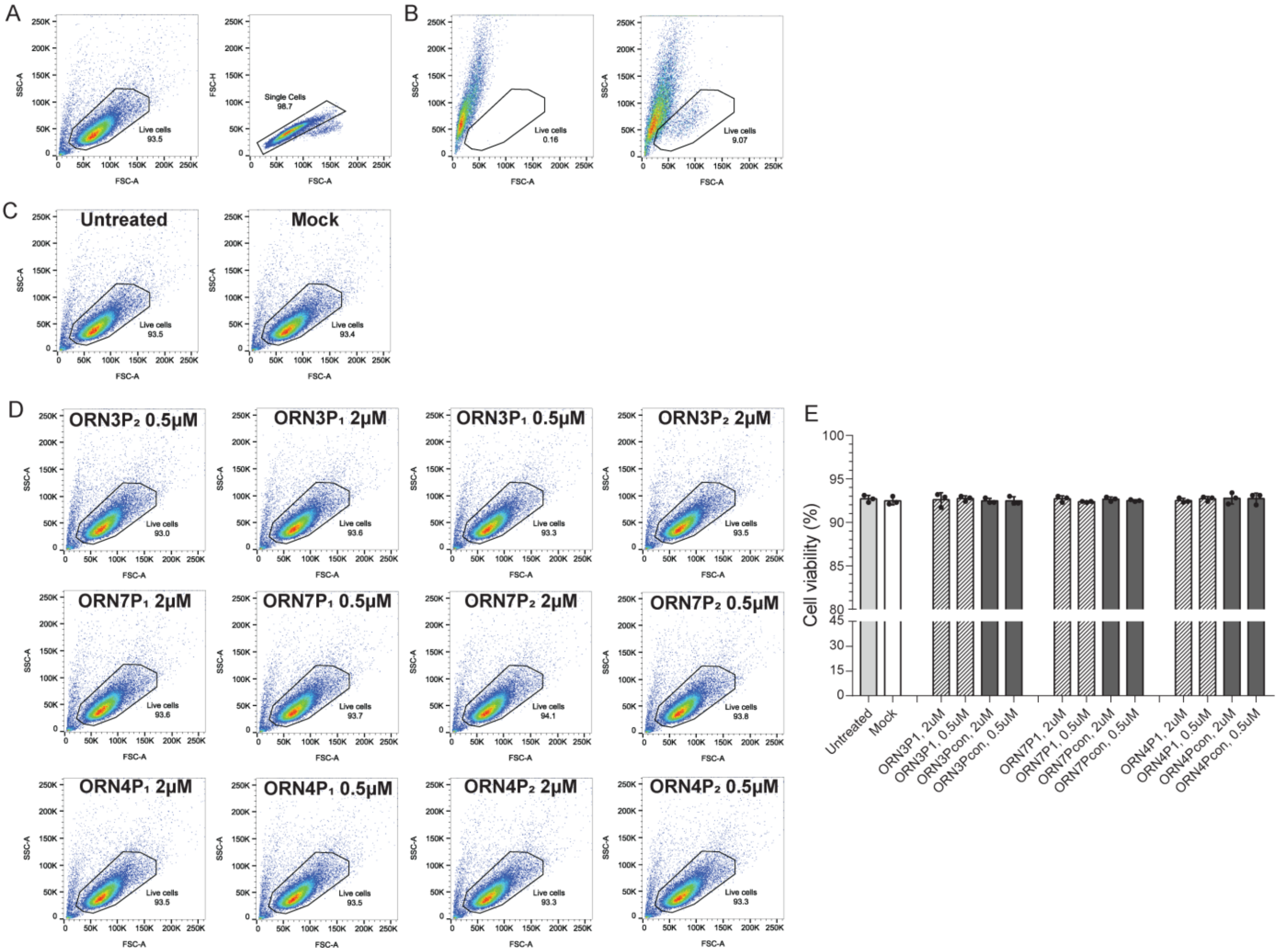

Figure S11. Cytotoxicity of RNA-PROTACs in K562 cells by fluorescence-activated flow cytometry (FACS). A) Gating strategy for the calculation of cell viability. Single, living cells were gated based on forward scatter (FSC-A) and exclusion of cell doublets. Cells in the area defined by the black boundaries are live cells. B) Chlorpromazine or gefinitib-treated cells (150 µM) were used as toxicity positive controls. C) and D) Representative FACS dot plots of cells treated with compounds **ORN3P$_1$**, **ORN7P$_1$**, **ORN4P$_1$** and correspondent controls. 50000 cells were acquired per sample.

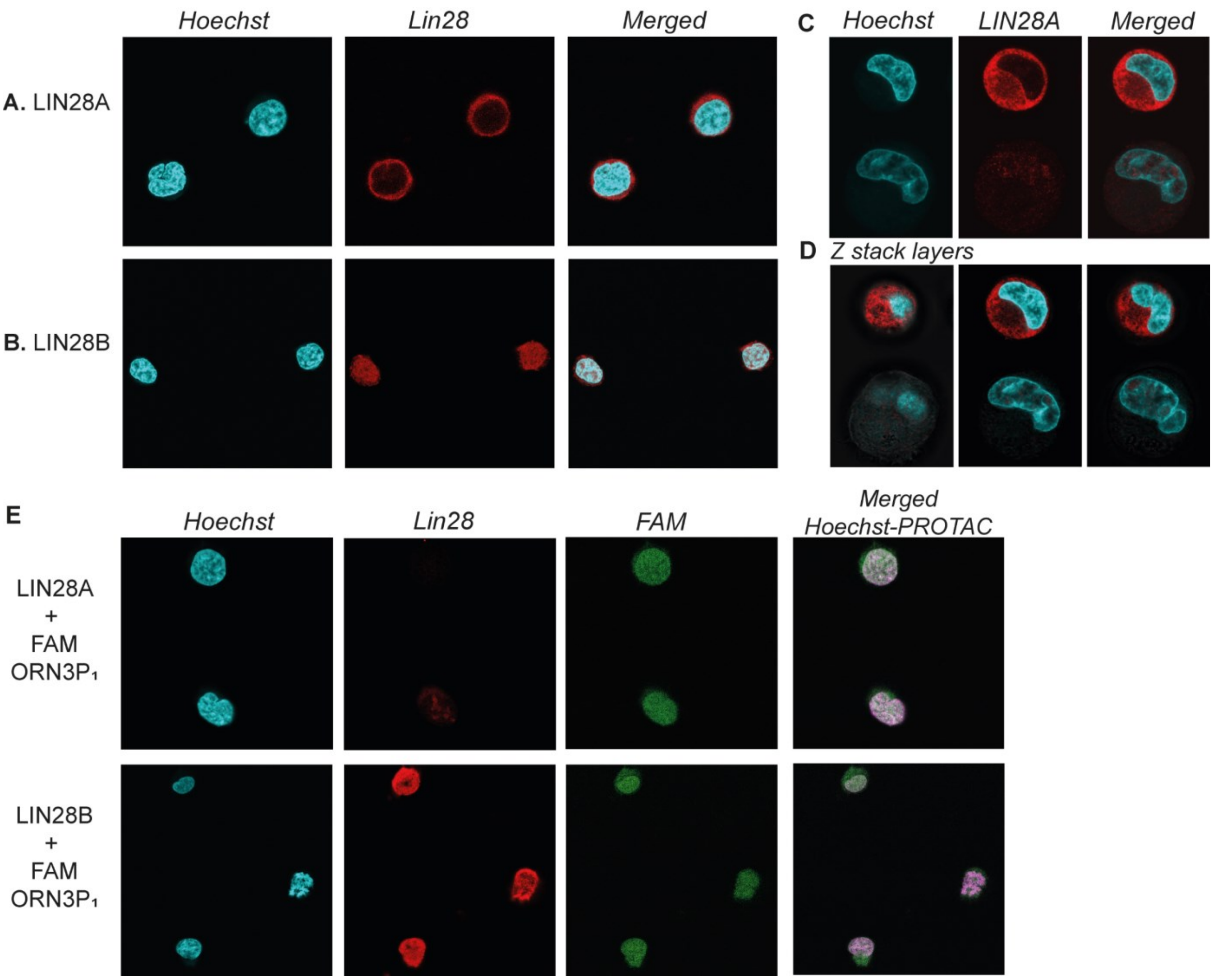

Figure S12. Confocal microscopy of K562 for cellular localization of LIN28 and the action of ORN3P$_1$.
Lin28A localizes in the cytoplasm (A) whereas LIN28B is abundant in the nuclei (B) of K562 cells; the Hoechst staining indicates the relatively large cytoplasm of K562 cells. C) Confocal microscopy of K562 for cellular localization of LIN28A (red); Hoechst staining indicates location of nuclei. D) Three different z-stack layers show the variation on the localization of LIN28A. E) Three-way staining of cells shows the presence of ORN3P$_1$, as well as Lin28A and Lin28B in the cytoplasm and nuclei of K562 cells; LIN28A is degraded by treatment with FAM labelled ORN3P$_1$ (top panel), whereas LIN28B is relatively unaffected by ORN3P$_1$ (bottom panel).

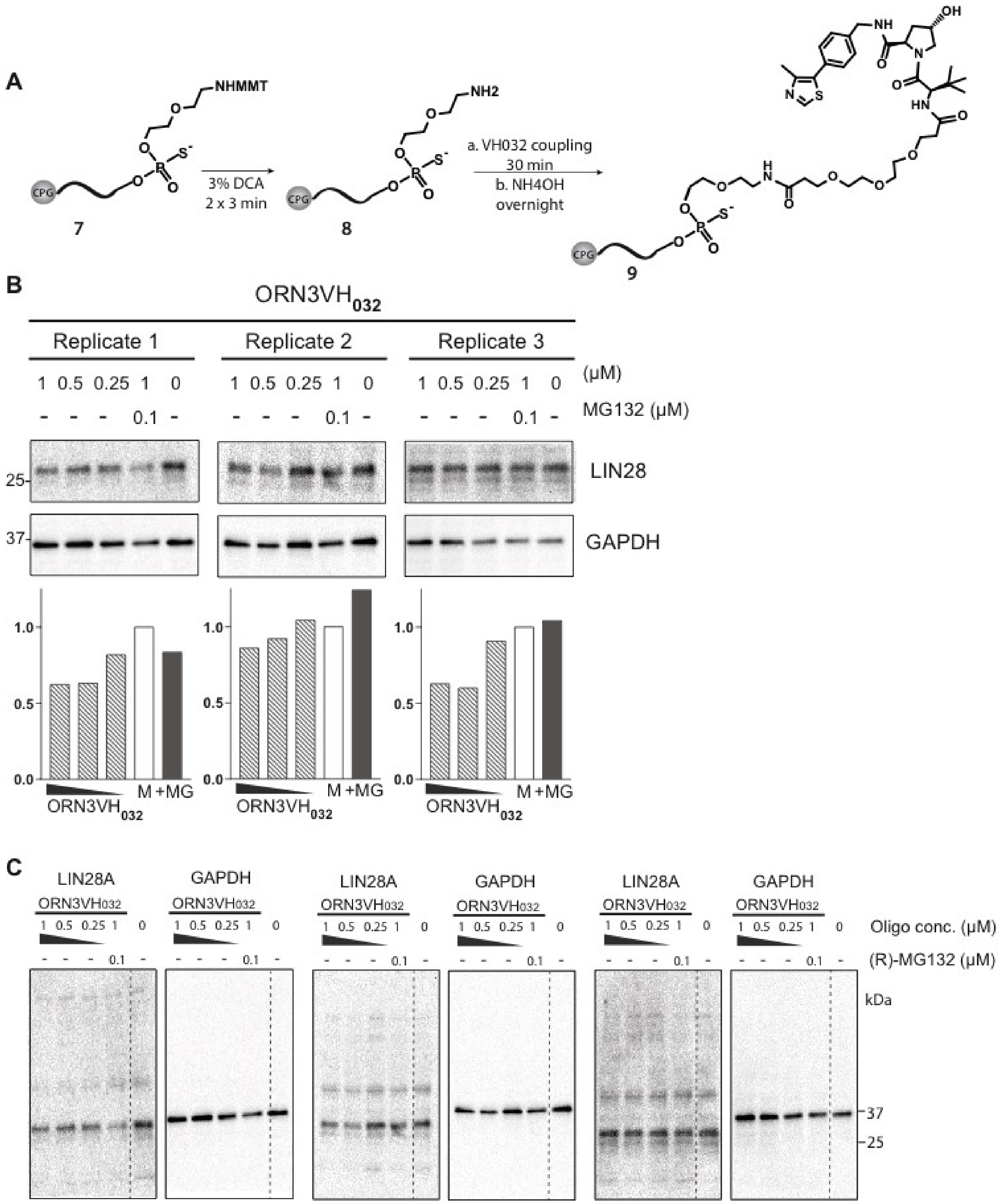

Figure S13. ORN3VH032 treatment of K562 cells: western blot membranes for LIN28A and GAPDH detection.
A) Synthesis scheme for **ORN3VH032**. B) Cut and C) uncut western blot and quantification of Lin28A from K562 cells after 24h treatment with **ORN3VH032**. Protein extracts were analyzed by immunoblotting with the indicated antibody.

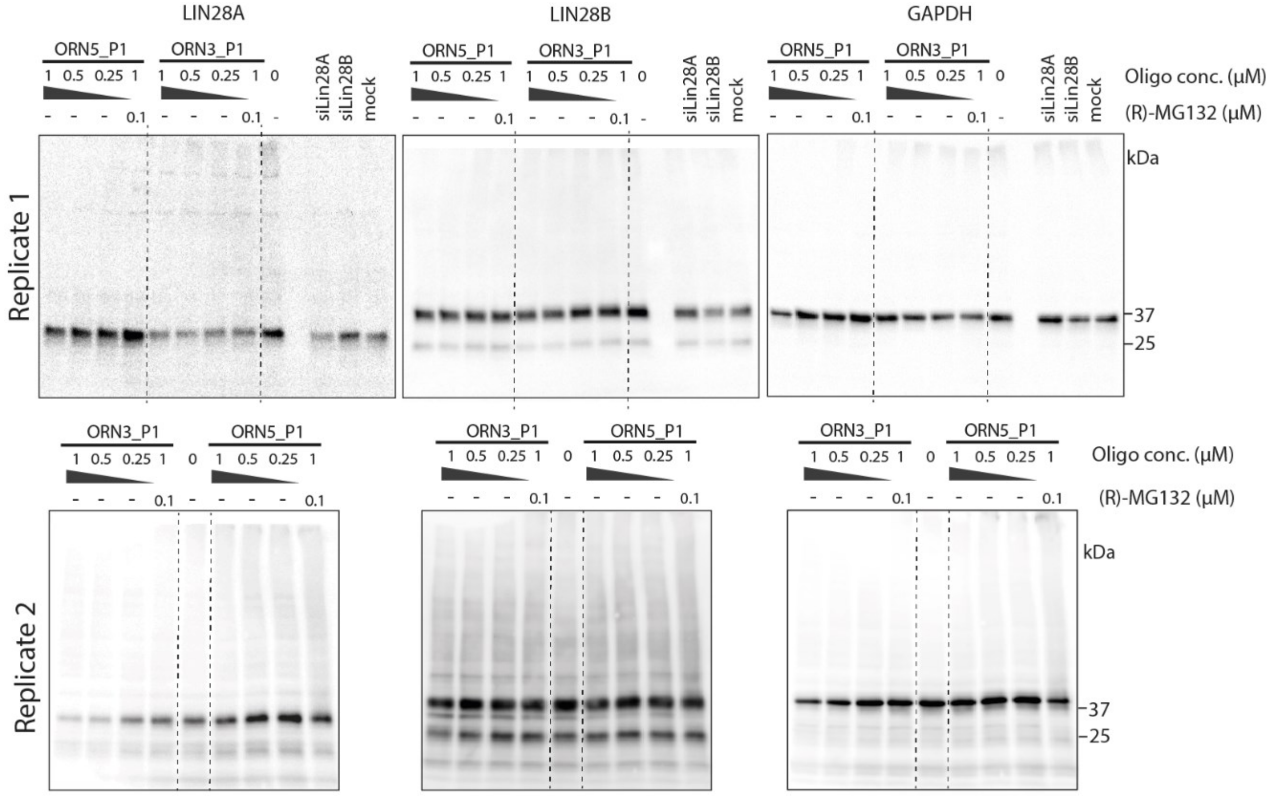

Figure S14. Uncropped western blot membranes for Lin28A, Lin28B, GAPDH detection in K562 cells. Western blot and quantification of Lin28A from K562 cells after treatment with **ORN3P$_1$** and **ORN5P$_1$** and corresponding controls. Protein extracts were analyzed by immunoblotting with the indicated antibody. SiRNAs treatments (siLin28A, siLin28B) are included solely to confirm that the bands on the western blots are Lin28A and Lin28B, respectfully. siRNAs for Lin28A and Lin28B were used to validate the antibody used for WB, ELISA and confocal experiments.

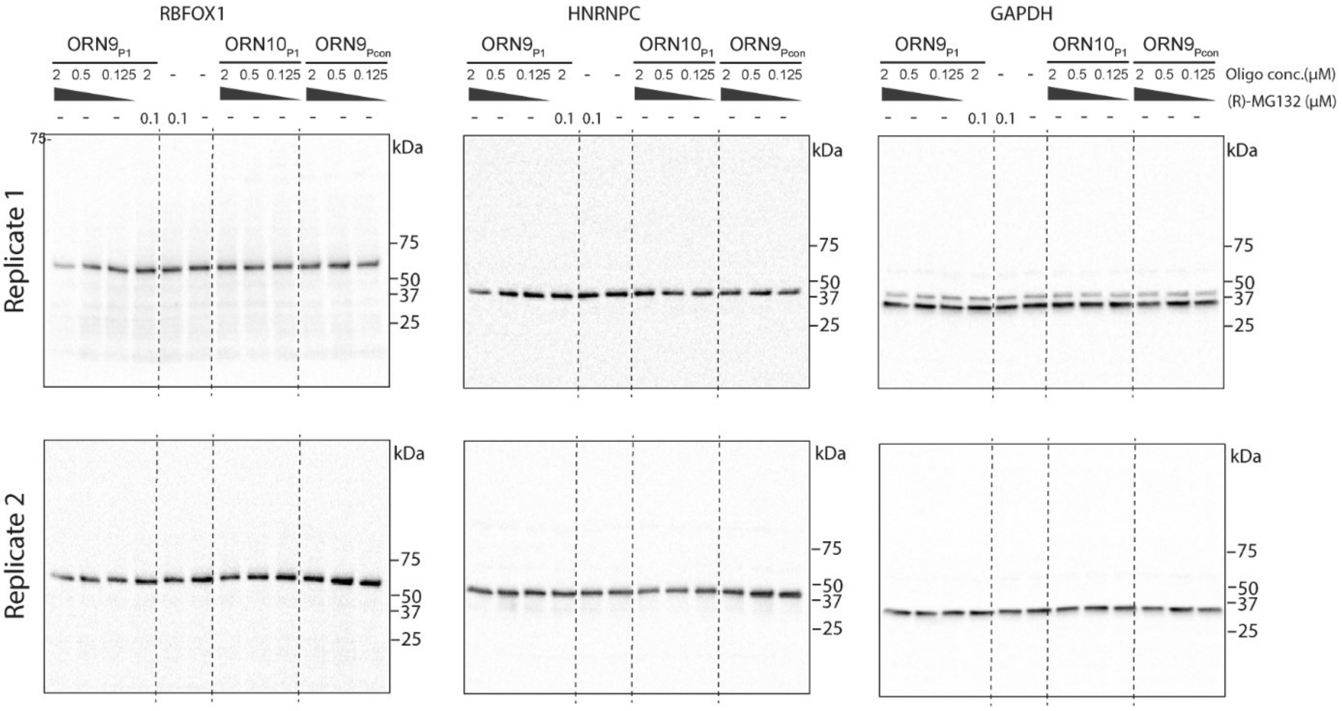

Figure S15. Uncropped western blot membranes for RBFOX1, HNRNPC, GAPDH detection in HEK293 cells. Western blot and quantification of Lin28A from HEK293 cells after 48h treatment with **ORN9P$_1$**, **ORN10P$_1$** and corresponding scramble ORN9P$_2$. Protein extracts were analyzed by immunoblotting with the indicated antibody.

REFERENCES

[1] H. Towbin, et al., *Nucleic Acids Research* **2012**, *41*, e47-e47.
